# Radioactive ion beam range adaptation in mouse tumors using in-beam PET

**DOI:** 10.1038/s43856-026-01786-1

**Published:** 2026-07-14

**Authors:** Martina Moglioni, Tamara Vitacchio, Francesco Evangelista, Munetaka Nitta, Daria Boscolo, Giulio Lovatti, Olga Sokol, Emma Haettner, Sivaji Purushothaman, Walter Tinganelli, Christian Graeff, Uli Weber, Christoph Schuy, Andreas Bückner, Gabriele Corbetta, Leonard Doyle, Lennart Volz, Maria Chiara Martire, Tim Wagner, Alexander Helm, Collin Werkheiser, Daria Kostyleva, Rinku Prajapat, Suraj Kumar Singh, Elena Rocco, Jonathan Bortfeldt, Peter G. Thirolf, Christoph Scheidenberger, Katia Parodi, Marco Durante

**Affiliations:** 1 GSI Helmholtzzentrum für Schwerionenforschung, Darmstadt, Germany; 2 Department of Medical Physics, Ludwig-Maximilians-Universität München (LMU), Munich, Germany; 3 Department of Electrical Engineering and Information Technology (ETIT), Technische Universität Darmstadt, Darmstadt, Germany; 4 Life Science Engineering Faculty, Technische Hochschule Mittelhessen, Gießen, Germany; 5 Institute of Condensed Matter Physics, Technische Universität Darmstadt, Darmstadt, Germany; 6 Institute of Physics, Justus-Liebig-Universität Gießen, Gießen, Germany; 7 Helmholtz Research Academy Hesse for FAIR (HFHF), GSI Helmholtz Center for Heavy Ion Research, Campus Giessen, 35392 Giessen, Germany; 8 Department of Physics “Ettore Pancini”, University Federico II, Naples, Italy

**Keywords:** Radiotherapy, Radiotherapy, Positron-emission tomography

## Abstract

**Background:**

Treatment adaptation is particularly critical in particle therapy, where even small range deviations can compromise target coverage or lead to unintended dose delivered to surrounding healthy tissues. In-beam positron emission tomography (PET) has emerged as a promising approach for range verification during irradiation with protons or stable carbon ion beams. However, its clinical use is limited by low signal-to-noise ratio and by the spatial mismatch between activity and dose distributions, reducing verification accuracy and limiting timely intervention.

**Methods:**

We used radioactive ion beams for real-time range adaptation in 10 weeks old C3H/ HeNRj female mice bearing LM8-osteosarcoma tumors. Three ^11^C beam range settings were planned: short (S), right (R), and long (L). The range of a collimated monoenergetic probing beam was monitored in real-time with the SIRMIO in-beam PET scanner by tracking the activity peak along the beam path, while range adaptation was achieved with a remotely controlled range shifter. Each plan (S, R and L) was also delivered, and tumor growth, toxicity assays, and histological analyses were performed to evaluate each treatment outcomes.

**Results:**

Dynamic repositioning of the ^11^C beam produced spatially resolved PET signals that correlated with distinct biological outcomes. Toxicity was observed only in the L group, while adequate tumor coverage was achieved in both R and L groups. In contrast, the S group showed continued tumor growth.

**Conclusions:**

We provide the first demonstration that in-beam imaging of radioactive ion beams can enable real-time range-guided radiotherapy in a living organism. These findings establish radioactive ion beams as a promising platform for precision range-guided particle therapy.

## Plain language summary

This study explores the use of radioactive ion beams to improve the precision of particle therapy through real-time PET imaging. In a mouse model of osteosarcoma, therapeutic ¹¹C ion beams were visualized during irradiation with a small-animal PET scanner, enabling sub-millimetric monitoring of beam range. Three beam depths were tested: too short, optimal, and too long. PET images acquired during treatment allowed rapid adjustment of beam penetration depth within the same irradiation session to ensure accurate tumor targeting. The biological outcomes matched the imaging results: insufficient range led to tumor regrowth, while excessive range caused damage to healthy tissues. This work provides the first demonstration of real-time range-guided radiotherapy using radioactive ion beams in a living organism.

## Abstract (web summary)

The authors investigate real-time range-guided particle therapy using radioactive ion beams, visualized by in-beam PET, in a mouse osteosarcoma model. Dynamic beam adaptation during irradiation achieves sub-millimetric targeting precision and enables accurate tumor coverage while minimizing damage to surrounding tissues.

## Introduction

Modern image-guided radiotherapy relies on high-resolution imaging by computed tomography (CT) or magnetic resonance imaging (MRI) to visualize the tumor in the patient before the treatment^1^. These images form the basis for treatment planning, aiming to maximize tumor destruction while sparing healthy tissue during the treatment. However, such pre-treatment images do not represent the patient’s anatomy at the exact time of irradiation or during beam delivery. In fact, the full treatment is usually delivered over several daily fractions, and both tumors and surrounding healthy organs can move, with these anatomical changes occurring over timescales ranging from sub-seconds (intra-fraction) to several hours (inter-fraction). Almost 30 years ago, it was first proposed that monitoring anatomical variations could allow for re-optimization of the treatment plan either prior to or during delivery, leading to improved precision and reduced toxicity in a highly personalized treatment^2^. This concept underpins adaptive radiotherapy, which is increasingly implemented in conventional X-ray-based radiotherapy, supported by advances in fast imaging and artificial intelligence (AI)^3^. Adaptive strategies are even more critical in radiotherapy with accelerated charged particles, such as protons or carbon ions^4^. In fact, while particle therapy offers physical and biological advantages compared to X-rays^5^, it is more susceptible to anatomical variations that significantly degrade the expected dose distribution in the patient^6^. Without proper inter- and intra-fractional adaptation, the precision benefits of charged particle therapy may be lost, resulting in unintended dose to healthy tissue and/or insufficient dose to the tumor^7^.

Adaptive radiotherapy is typically classified into three categories: offline, online, and real-time^8^. While in offline adaptive radiotherapy the treatment plan is modified using images acquired in previous sessions, online adaptation involves re-planning using images registered with the patient in the same position of the beam delivery shortly before the treatment, usually by cone-beam CT (CBCT), in-room CT or MRI. However, those imaging techniques do not provide information on potential dose deviations during irradiation, although such deviations can be indirectly estimated via image-based dose recalculation performed either prior to or following the treatment session. Therefore, imaging techniques which allow for the prompt detection of dose discrepancies during beam delivery (in-beam) are highly desirable, as they can support real-time patient-tailored treatment adaptation^9,10^. Real-time adaptation requires ultra-fast plan update during the beam delivery^11^, a capability currently in development for MRI-linacs in X-ray therapy^12^. It is important to note that the term *“real-time”* is widely used in the literature; however, given the time required for image acquisition, reconstruction, and plan recalculation, the procedure is never really in real-time. In this paper, we will use the word “real-time” to describe dynamical and fast range monitoring and adaptation during irradiation. Online adaptive proton therapy has been already successfully tested in clinical settings^13,14^ but this is not the case for real-time particle therapy. To date, only in silico dose reconstruction synchronized with treatment delivery has been demonstrated for carbon ion beams^15,16^.

In-beam positron emission tomography (PET) is one of the most promising strategies to verify dose delivery in real-time during particle therapy^17,18^. In ^12^C-ion therapy, in-beam PET imaging measures the activity distribution of the β⁺-emitting isotopes, primarily ^11^C (t_1/2_ = 20.33 min) produced by nuclear fragmentation of the therapeutic stable ^12^C beam within the patient body, while in proton therapy the positron-emitting radioisotopes (e.g., ^15^O and ^13^N) are produced by target fragmentation. Recent studies have demonstrated the potential to detect proton or ^12^C-ion dose deviations due to morphological changes in patients through an extensive offline analysis and post-processing of previously acquired in-beam PET data^19–22^. Further investigation is directed toward correlating dose shifts along the beam axis with the range shifts detected in the PET images^23^, and at developing methods, including AI, for in-beam dose reconstruction using PET data acquired during proton therapy treatments^24^. However, presently those studies are limited to phantom experiments^25^ or based on simulations^26^. All in-beam PET measurements are hampered by the low counting rate of the activity signal^21^, limited resolution and image artifacts^27^, and the significant spatial mismatch between the activity peak of the PET distribution and the Bragg peak of the primary beam, making practical applications in the clinics difficult^28^.

The use of radioactive ion beams, as opposed to stable beams, has the potential to overcome many of these limitations, because the shift between activity and dose peaks is reduced^29^ and the signal-to-noise ratio increases by approximately an order of magnitude^30^. Exotic beams are a key tool for modern studies of nuclei at extreme conditions in nuclear physics^31,32^, and for many years they have been proposed for cancer radiotherapy^33^, but application was always limited by the low secondary beam intensity. In the BARB (Biological Applications of Radioactive Ion Beams) project, we aimed at exploiting the high-intensity of the GSI/FAIR facility and its fragment separator (FRS) to give a first demonstration of pre-clinical tumor treatment with radioactive beams^34^. Within BARB, we have successfully treated solid tumors in the neck of C3H mice using a ^11^C-beam at GSI^35^ with simultaneous beam imaging acquired by the Small animal proton Irradiator for Research in Molecular Image-guided radiation Oncology (SIRMIO) PET scanner^36^. Even if the tumor was very close to the spinal cord, no significant radiation myelopathy was induced. This experiment was a proof-of-principle of the use of radioactive beams for image-guided particle therapy^37^, showing a strong spatial correlation between the PET activity peak and the 80% distal dose fall-off across all mice. Such a level of agreement is challenging to achieve with conventional stable ¹²C ion or proton beams. In these cases, intrinsic limitations of in-beam PET—such as lower signal-to-noise ratio and significant reduced spatial correlation between activation and dose deposition—can hinder precise range verification during irradiation. Previous work on range-guided adaptive proton therapy demonstrated the feasibility of online range verification using in-beam PET imaging of mid-range probing layers during pencil beam scanning delivery, achieving range-shift estimation within 60 s of PET acquisition and with 0.5 mm uncertainties^38^. However, these studies were limited to phantom-based single-field uniform dose proton irradiations with range shifts of approximately ± 6.8 mm and relied primarily on indirect estimation of global range deviations from mid-range probing spots, rather than direct monitoring of the clinically relevant distal dose fall-off. These limitations motivate the need for more robust real-time range monitoring.

**Fig. 1:**
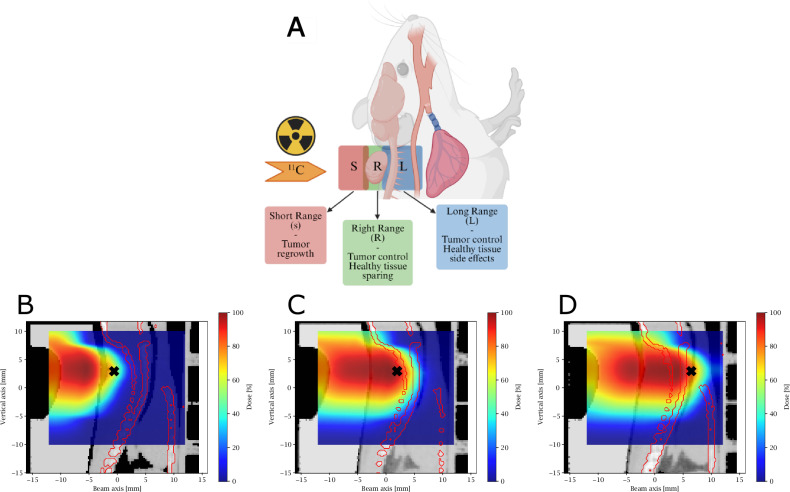
Goldilocks range. **A** Conceptual representation of the study and experimental groups. The ^11^C-beam is used for three different treatment settings: Short range (S, red area), Right range (R, green area), and Long range (L, blue area). The working hypothesis is that the S range treatment will not cover the tumor, hence not controlling it, while in the L range the tumor will be treated, but there will be healthy tissue side effects caused by radiation. In the R range instead, the Goldilocks range, the tumor is eradicated and the healthy tissue spared. Created with BioRender.com. **B** FLUKA simulations showing the planned ^11^C-ion dose (in percentage of the maximum dose value) distribution in the SARRP scan of a mouse in the sagittal view for the Short (S) experiment. Range at 60% of the maximum value (R60) is indicated with a black cross. **C** Same as in **B** for the Goldilocks (R) experiment, overlayed on the CBCT scan of a different mouse. The cross marks the range at 95% of the maximum value (R95). **D** Same as in **B** and **C** but now for the L mode experiment, overlayed on the CBCT scan of a mouse part of the L group. The cross marks the range at 80% of the maximum value (R80). Figures (**B**–**D**) were reoriented to be consistent with the reference frame of the PET scanner.

Here, we investigate the hypothesis that radioactive ion beams may provide an effective platform for real-time range-guided particle therapy through in-beam PET-based range verification with sub-millimetric precision ( < 0.5 mm). Using our osteosarcoma-C3H mouse model, we produced CT images of the animal shortly before irradiation in the same vertical position used for the exposure to the beam. We have then visualized in real-time the therapeutic ^11^C beam penetrating along the axis perpendicular to the mouse spine with the SIRMIO in-beam PET scanner. Three different ^11^C beam depth settings were planned. In the first test group, the beam was planned to stop at the beginning of the tumor (“short” range; S); in the second - to cover the whole tumor while sparing healthy tissues behind it (“right” range; R); and in the third - to traverse tumor, spinal cord and stop in the esophagus (“long” range; L). A representative illustration of the three beam positions is shown in Fig. 1A, while simulated doses can be found in Fig. 1B, C, D for the S, R and L ranges, respectively. This “aim-and-shoot” approach is used to irradiate the “just right” volume, i.e., to find the “Goldilocks range” with in-beam PET. Our goal is to demonstrate dynamic range adaptation during irradiation based on in-beam PET data. Beam penetration depth is verified and iteratively adjusted within the same irradiation session to match the target depth, ensuring adequate tumor coverage. Each treatment plan was also delivered individually, and range verification was performed to later assess the correlation between range accuracy and biological outcome under conditions of under-shooting, optimal targeting, and over-shooting of the tumor volume. In this experimental setup, toxicity was expected only in the L-group, while tumor control was achieved in the R and L positions.

## Methods

### Radioactive ion beam production and delivery

The ^11^C beam was generated via in-flight fragmentation. A 300 MeV/u ^12^C primary beam from the 18 Tm heavy ion synchrotron (Schwerionensynchrotron; SIS18) was directed onto an 8.045 g/cm² beryllium target at the FRS^39^, where peripheral nuclear reactions stripped one or more nucleons, producing a range of lighter isotopes. Isotopic purification of ^11^C was achieved through a combination of magnetic rigidity selection and energy-loss separation in a wedge-shaped degrader positioned at the FRS central focal plane. This method yielded a beam with a purity of approximately 98%^35^. For the current experiment, the purified beam was transported via the connecting beamline to Cave M as described in detail elsewhere^40^. The primary beam, with an intensity of 1.7·10^10^ particles/spill inside the SIS18 ring yielded a 210.8 MeV/u ^11^C beam with an intensity of 3.5·10^6^ particles/spill in the experimental room. Beam extraction lasted 0.25 seconds with a spill pause of 1.75 s to optimize online PET acquisition. All irradiations were performed in Cave M: we will indicate the extracted beam in air as “monoenergetic” or “pristine” beam, even if it has a significant momentum spread Δp/p = 0.6%. Beam parameters are summarized in Table 1.

**Table 1:** Summary of the beam parameters used in the experiments

Spill length	200 ms
Duty cycle	12%
^12^C beam energy	300 MeV/u
^11^C beam energy at isocenter	210.8 MeV/u
^12^C intensity in SIS18	1.7·10^10^ particles/spill
^11^C intensity in Cave M	3.5·10^6^ particles/spill
Beamspot shape	Horizontal direction: 1.813 cm FWHM Vertical direction: 1.365 cm FWHM
Momentum spread	σ = 0.00556 GeV/c·u, truncated at ± 2σ

**Fig. 2:**
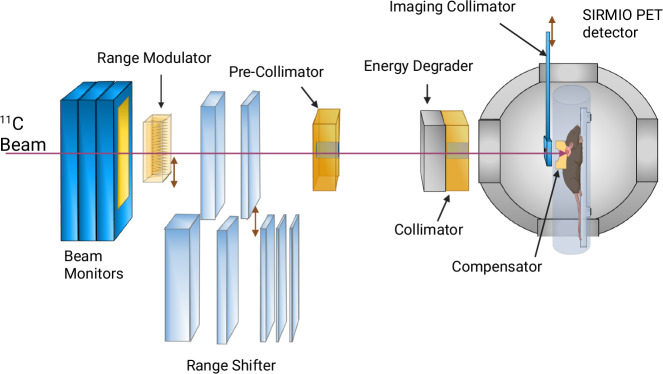
Experimental beamline. Schematic representation of the experimental beamline setup. The mice were positioned vertically within the SIRMIO PET scanner for irradiation, while a sequence of passive components modified the beam to match the treatment volume. Calibrated large-plate ionization chambers served as beam monitors for the pristine ^11^C beam. A 2D range modulator was used to produce a 1.2 cm SOBP in water. To tune the penetration depth to the approximate tumor location, plastic and aluminum degraders (7.0 mm WET polyethylene and 51.5 mm WET aluminum) were introduced in the beamline. Two brass collimators restricted the lateral beam spread, blocking regions that would not contribute to the target dose. Additionally, a custom plastic mouse collar, fixed to the animal bed, functioned as a compensator by partially attenuating the beam outside the CTV and shaping the distal SOBP edge to follow the target outline. The fine adjustments of the distal edge of the SOBP for the three irradiation configurations were done by using a range shifter introducing polyethylene absorbers for a total of 14.4 mm WET, 8 mm WET, and 5.1 mm WET for the S, R and L range treatment groups, respectively. An additional probing collimator with a 3 mm aperture was mounted in the SIRMIO PET scanner. This last component was used with a monoenergetic ^11^C probe beam to verify the range before delivering the complete treatment. Created with BioRender.com.

The beamline setup was adapted from the previous study^35^: the beam was shaped by a passive system for conformal dose deposition including a range modulator, plastic absorbers, aluminum degraders, collimators, and a compensator (see Fig. 2). The mice were treated in a vertical position inside the SIRMIO PET scanner (see section PET imaging for details), fixed with a dedicated compensator onto a custom-made holder in a way that the center of the generalized clinical target volume (CTV) was located at the isocenter position of the PET scanner. In particular, the inner structure of the compensator was anatomically shaped to ensure reproducible mouse positioning, while the outer surface included a 5 mm-deep dimple corresponding to the WET up to the distal edge of a universal target region (named generalized CTV) that accounts for small variations in tumor size and location between individual animals.

A 2D range modulator (2DRM) was used to generate a 1.2 cm Spread-Out Bragg Peak (SOBP) from the monoenergetic beam. To enable rapid switching between monoenergetic and SOBP delivery during treatments, the 2DRM was mounted on a remotely controlled range shifter.

Plastic and aluminum degraders were employed to reduce the beam energy and to position the Bragg peak approximately at the mouse position. Distal fall-off adjustments for the three irradiation modes (S, R, L) were achieved by selecting combinations of plastic plates in the remotely controlled range shifter. Owing to the discrete set of absorber plate thicknesses available and the water equivalent thickness (WET) of the 2DRM, an exact match in penetration depth between pristine and SOBP irradiations could not always be achieved for the same irradiation mode. Nonetheless, the resulting discrepancy was negligible: the maximum depth deviation between the SOBP and probe configurations was 0.40 mm.

In comparison to the previous setup^35^, a key upgrade was a stainless-steel collimator (3 mm aperture) positioned along the beam path and used with a monoenergetic ¹¹C probe beam to verify the range prior to the delivery of the full target dose. To minimize the scattering, the collimator was mounted in the SIRMIO PET scanner, as close as possible to the mouse holder. A motorized stage allowed a fast collimator extraction before the delivery of the treatment SOBP irradiation. In the presence of this collimator, based on FLUKA simulations (see Treatment Planning and Irradiation section), we estimated an effective transmission factor of approximately 1% through the full beamline up to the compensator.

### Dosimetry

**Fig. 3:**
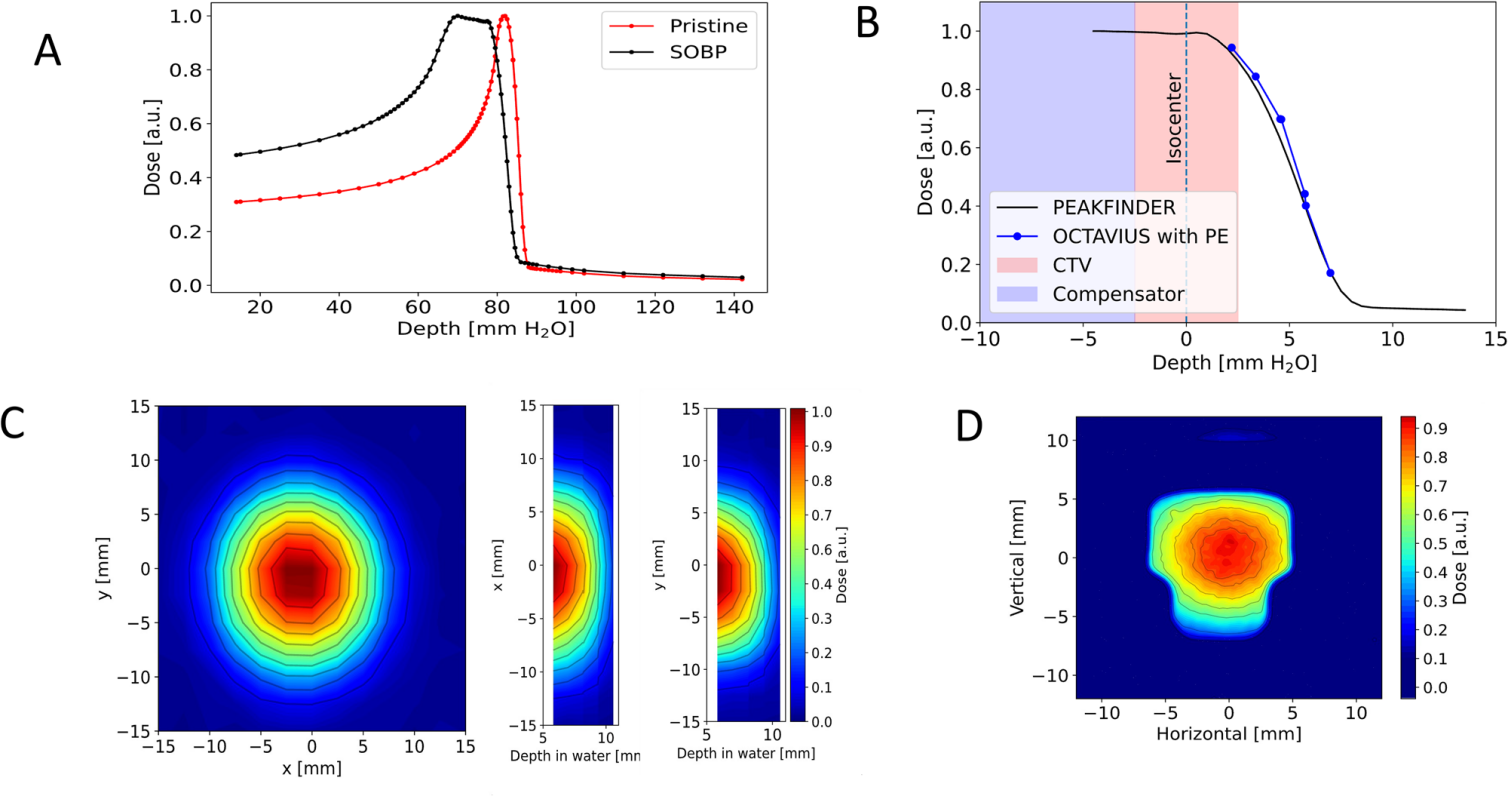
Beam longitudinal and lateral distributions. **A** Laterally integrated depth–dose profiles in water for the monoenergetic (pristine) beam (red) and SOBP (black) measured with PEAKFINDER™ without additional beam modifiers. **B** (Solid black) Depth–dose distribution measured with the PEAKFINDER™ after beam attenuation (7.0 mm WET polyethylene and 51.5 mm WET aluminum) and collimation. (Blue dots) Depth–dose distribution for the R treatment beamline configuration measured by the central chamber of the PTW OCTAVIUS 1600 XDR (minimum measurable water-equivalent depth: 2.2 mm WET from the isocenter position). Different depths in water were obtained by adding PMMA slabs of varying thicknesses in front of the detector. All curves are plotted in the PET isocenter reference system. See Supplementary Figs. 2, 3 for setup schematics. CTV and collar regions are marked by pink and blue bands. **C** Beam spot at the distal edge of the CTV (2.2 mm WET from isocenter) and horizontal/vertical 2D dose distributions in the central plane along the beam direction measured with OCTAVIUS 1600 XDR setup described above. **D** Lateral dose distribution at the CTV center acquired with the complete BARB beamline operating in the R modality. Measurements were taken with a Gafchromic™ EBT3 film inserted into a custom-designed compensator fixed on the mouse holder.

In Cave M, beam fluence was monitored with large parallel-plate ionization chambers^41^. Beam modulation, characterization, and dosimetry followed the approach of previous studies^35^. A PTW PEAKFINDER™ system (PTW Freiburg, Germany) and an in-house water phantom^42^ equipped with an OCTAVIUS 1600 XDR detector (PTW Freiburg, Germany) were used to measure lateral profiles and depth-dose distributions in water. Figure 3A shows the measured depth–dose distributions for pristine beam and SOBP in water. Measured beam spot and 2D depth dose distributions for the pristine irradiation are shown in Supplementary Fig. 1. Simulated WET of all materials were also verified with the PEAKFINDER™ system.

After the setup was built and aligned, 1D and 3D depth dose distributions were then measured again at the mouse irradiation position (Fig. 3B, C). For the 3D dose distributions, as the residual range for mice irradiation was shorter than the minimal WET of the water phantom, the OCTAVIUS 1600 XDR detector was mounted in a plastic holder and a series variable-thickness PMMA plates with a WET matching the distal edge of the CTV were used as bolus plates in front of the detector. See Supplementary Figs. 2, 3 for the dosimetry setups at the tumor position.

Absolute dosimetry at the tumor site was performed using a PTW TM31023 small-volume pinpoint ionization chamber in a custom holder to align the sensitive volume with tumor depth. The measured dose established a setup-specific monitor unit calibration for plan scaling. Lateral beam profiles at the isocenter after compensation were verified with Gafchomic^TM^ EBT3 films (Fig. 3D), using a dedicated compensator holding radiographic films at a WET corresponding to the CTV isocenter position.

### Treatment planning and Irradiation

The previously developed FLUKA^43^ Monte Carlo tool for the BARB experiment^35^ and the available CBCT scans of animals in vertical position with the customized setup-specific CT number to stopping power calibration^44^ and the same compensator for the previous experiment were used to support the treatment planning workflow and the design of new beamline components. Inter-mouse anatomical variability in the treatment position was found to be within the spatial resolution of the SARRP system and therefore did not introduce significant differences among the three irradiation configurations and the resulting treatment plans. Simulations were conducted by using the FLUKA v2024.1.2 with the flair GUI v2.3-0 version and by activating the HADRONTherapy, COALESCEnce, and IONSPLIT cards and a customized USERROUTINE for reproducing the experimental SOBP^45^. Primary particle numbers were set to keep statistical uncertainty below 1%.

Simulations were conducted first to design the collimator for the probe beam, to define the collection of range adsorbers necessary to obtain the accurate penetration depth corresponding to the three ranges (S, R, L) in mice and to ensure accurate beam delivery. After the experiment beam model characterization (see Table 1) and final treatment verification were performed.

For the S mode, the beam was planned to stop at the proximal edge of the CTV, precisely at the range corresponding to 60% of the SOBP dose (R60; 0.3 mm WET inside the mouse body). The Goldilocks range experiment (R) was designed to cover the whole CTV depth with an R95 at 5 mm WET inside the mouse. Finally, in the L group, the beam was planned to traverse the tumor and spine, with the R80 positioned in the esophagus, located at a depth of approximately 9.2 mm WET in the mouse body. Figure 1B–D show the simulated SOBP reaching the planned ranges, S, R and L, respectively, in a mouse with respect to the isocenter position in the beamline. Each range is highlighted with a black cross. From our previous experiments, we established that the position of the 80% distal dose fall-off (R80) can be derived from the PET activity peak, enabling us to perform accurate beam range determination in each irradiation mode. Further estimation of R60 and R95 reference depths were derived from monoenergetic beam dosimetry in water, where they were found to lie approximately +1 mm and −1.5 mm relative to R80, respectively. We further verified through simulations that these millimetric offsets are also reproduced in the activity distributions, which consistently exhibit the corresponding spatial shifts along the beam axis in the PET images.

This spatial relationship was used for initial verification of each beam range (S, R, or L) by observing the in-beam PET activity distributions obtained during the irradiation with the collimated monoenergetic ^11^C beam, overlapping them with the CBCT scans of the respective animals. Dose delivered in the spot was 5 Gy. The choice was based on our previous study^35^ where we demonstrated that such a dose does not induce significant tumor damage when delivered in a single fraction. Therefore, such probing irradiation can be considered biologically sub-therapeutic and not expected to bias the subsequent treatment response in the present experiment. The dose rate was approximately 1 Gy/min, corresponding to about 5 min total beam delivery, with 3.65·10^5^ particles/spot through two beam spots in the y direction ( ± 3 mm) repeated for 500 times for the probing irradiation.

For each run, the in-beam PET images were produced approximately every 25 seconds during irradiation, providing fast feedback on the beam range^25,46^. After confirming the range, we irradiated the desired volume with a 20 Gy SOBP plan whose distal edge was falling at the position of the monoenergetic Bragg peak used as probe.

**Fig. 4:**
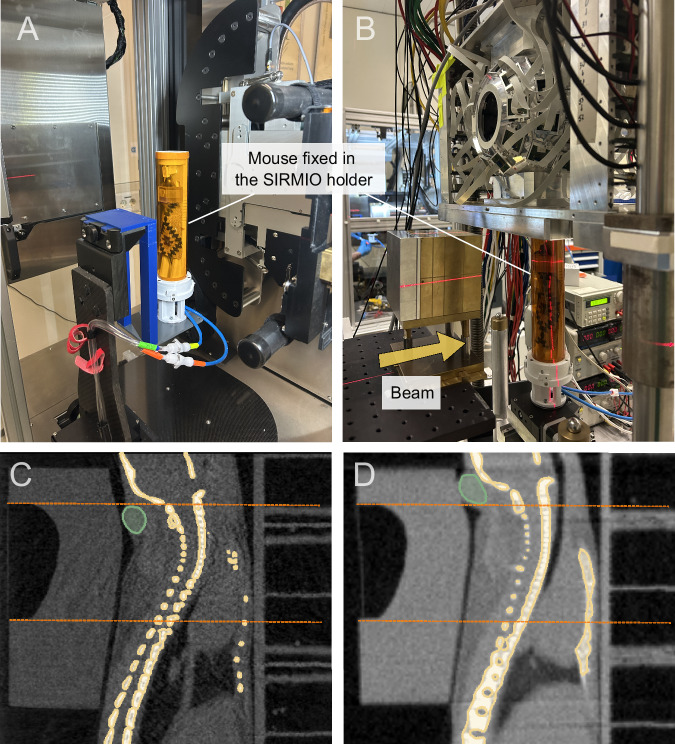
Pre-irradiation animal imaging. Vertical positioning of the mouse first inside the SARRP machine (**A**) for the CBCT scan acquisition in treatment condition and then inside the SIRMIO scanner (**B**) for irradiation and PET image acquisition. Images **C** and **D** show the change in the mouse anatomy that can occur due to the repositioning between the ‘diagnostic’ scan acquisition (μCT, horizontal) and treatment. Segmentations of the tumor (green) and skeleton (yellow), contoured on the horizontal μCT scan acquired one day before the irradiation (**C**) and on a vertical CBCT scan acquired in the treatment position immediately before the irradiation (**D**), are shown. Dashed orange lines indicate the lateral extent of the irradiation area. On the ‘diagnostic’ μCT scan the tumor is safely in the field, while during the actual treatment delivery it moved towards the skull. Eventually, this animal had to be excluded from the experiment.

Immediately before the start of the treatment, we acquired a CBCT of each mouse using the Small Animal Radiation Research Platform (SARRP), see Fig. 4A and section CT imaging – Vertical imaging, to get the anatomical image of the animal in exactly the same vertical position used for irradiation (Fig. 4B). This was possible due to using the multi-purpose holder compatible with both setups, avoiding, and repositioning the animal between the CBCT scan and irradiation.

Our workflow follows a range-guided radiotherapy paradigm, in which imaging acquired immediately prior to treatment was used for real-time in-beam PETrange verification and adaptation. The importance of pre-treatment anatomical verification can be seen by comparing Fig. 4C, showing a mouse anatomy on a control μCT in horizontal position (see section CT imaging – Horizontal imaging) the day before the irradiation, to Fig. 4D showing the CBCT of the same animal at the time of irradiation. Clearly, the tumor is shifted away from the irradiation field. This would require not range adjustment (at least not in the first place) but instead a vertical adjustment of the mouse holder to bring the tumor in the irradiation field, which was not technically possible since the beamline setup was fixed. A total of three animals had to be excluded using pre-irradiation imaging for this reason. If such kind of tumor position discrepancy would have been observed in the BEV direction, this would have been the case for range adjustment.

### Animal model

The animal experiment conducted in this work has been approved by the German Federal Law under the approval of the Hessen Animal Ethics Committee (Project License DA17/2003) and was performed in accordance with national regulations for animal welfare. This study is reported in compliance with the ARRIVE 2.0 guidelines. All the animals used in the study were 10 weeks old C3H/ HeNRj female mice (*Mus musculus*, Janvier Labs, France) and were housed at GSI at standard conditions (non-SPF, 22 °C, 55% humidity, 12 h inverted light-dark cycle), with *ad libitum* access to conventional rodent food (Ssniff, Germany) and water. The mice were given a week of acclimatization, after which they were administered a subcutaneous injection of 20 µL of PBS buffer containing ~10^6^ syngeneic Dunn osteosarcoma LM8 cells (Riken BioResource Center, Japan) in the cervical area. The injections were performed under anesthesia (2% isoflurane mixed with air) inhaled via a face mask, to maintain consistency in the administration area. After 14 days, the majority of the tumors were visible and measurable both with the caliper and the µCT. Mice were divided in the following five groups: 9 control tumor-bearing mice (sham-irradiated, kept for 1 month), 7 “short range” irradiated animals (S, kept for 1 month), 8 “Goldilocks (right) range”, and 10 “long range” irradiated animals (R and L, respectively, kept for 6 months), and 8 tumor-free controls (sham-irradiated, kept for 6 months). Irradiations were performed fourteen days after the tumor injection. A total of 26 mice were irradiated with a dose rate of approximately 1.3 Gy/min in the CTV.

### CT imaging

#### Horizontal imaging – μCT

The data on the tumor growth and location was acquired via the μCT measurements with a VivaCT 80 scanner (SCANCO Medical AG, Switzerland) following the protocol described in ref. ^35^. One day before the ^11^C irradiation (to confirm the tumor presence and location) and then weekly for 28 days after the irradiation (to monitor the growth delay). Briefly, mice were positioned onto a horizontal holder with the geometry resembling the one of the SIRMIO bed and additionally immobilized using the compensator collar. The anesthesia was induced with isoflurane (3% for the induction, 1.5% for maintenance during the scan). The tube settings for all the scans were 45 kVp and 177 μA for the voltage and current, respectively, with an addition of a 0.1 mm aluminum filter. We have used the same concept of generalized CTV as in our previous work^35^ to delineate the universal target area accounting for the unavoidable slight variability in the tumor sizes and locations. The horizontal μCT was not used for treatment planning because anatomical variations in the of the tumor position and curvature of the spine^35^ were observed between the horizontal imaging configuration and the vertical positioning used during irradiation. Therefore, the following Vertical imaging – SARRP was adopted for treatment planning.

#### Vertical imaging – SARRP

To get the CT image of the animal in the actual treatment condition, we have utilized CBCT of the Small Animal Radiation Therapy Platform (SARRP, XStrahl, United States) located in Cave M next to the BARB beamline. We used a custom-designed adapter enabling the use of the same SIRMIO mouse holder for both imaging and irradiation avoiding the mouse repositioning between the two procedures (Fig. 4A, B). This way, the mouse was positioned on the SIRMIO bed, additionally immobilized with the compensator collar, and then the enclosed holder was first mounted in the SARRP for the vertical CT image acquisition, and then reinstalled in the beamline for ^11^C treatment. Throughout the procedure, animals were anesthetized with isoflurane. The scans were acquired using the default machine settings for the CBCT mode (70 kVp and 1 mA tube voltage and current, respectively, 0.15 mm copper filter, 180 projections per 180°) resulting in the images with a voxel size of 275 μm. After the scan was complete, the animal holder was extracted from the SARRP and mounted in the SIRMIO scanner for the following irradiation and PET image acquisition. Acquired images were immediately transferred for further analysis of measured PET distributions.

### PET imaging

All irradiation experiments were monitored online using a dedicated spherical in-beam PET scanner with 56 LYSO scintillation crystals with depth of interaction (DOI) capability for unprecedented sensitivity and spatial resolution^36,47^. The online image reconstruction refers to a reconstruction strategy in which data are continuously read from the digitizer in subsets defined over short acquisition windows. While new events are still being acquired, the incoming list-mode data are filtered, and the reconstruction algorithm (list-mode Maximum Likelihood Expectation Maximization, MLEM) is applied in parallel. The goal is to have both the updated list-mode system matrix contributions and the current MLEM iteration computed before the next data subset is ready to be integrated, thereby maintaining a continuously updated image estimate. For this study, data streams corresponding to acquisition times between 25 and 120 s were used to test different reconstruction configurations. The raw data were filtered with an energy window of 440–580 keV, corresponding to ±1σ obtained from a Gaussian fit of the photopeak. The coincidence time window was set to 20 ns. The image reconstruction was performed using MLEM with a voxel size of 0.5 × 0.4 × 0.4 mm³, corresponding to the lateral, vertical, and beam-direction dimensions, reflecting the different spatial resolution of the spherical in-beam PET scanner at the respective axes. At each MLEM iteration, the image was corrected using an experimentally determined, voxel-dependent sensitivity map and an additional background correction term accounting for the intrinsic radioactivity of ¹⁷⁶Lu present in the LYSO scintillators.

### Co-registration

The mouse position within the SARRP imager was co-registered with the PET imaging reference system using a three-step procedure:

#### Co-registration of the motorized stage to the PET system

A ^22^Na point source was attached to the mouse holder in a well-defined position with a collar of similar geometry as the mouse immobilization collar but with an insert that fix the ^22^Na source hold the source at the center of the CTV position, and several measurements were acquired while moving the holder through known stage positions. These measurements were used to determine the transformation between the 4D motorized stage coordinates (3D translation + rotation) and the PET image reference frame.

#### Alignment

After stage-to-PET co-registration, the mouse holder with the immobilization collar was mounted on the motorized stage. Alignment of the entire beamline, including the mouse holder, was performed in beam by inserting additional 1 mm collimators into the apertures of the existing collimators and pre-collimator, thereby restricting their effective aperture. If the collimating system was not correctly aligned, the beam would not reach the holder. The beam at the target position was imaged using radiographic film attached to a dedicated alignment collar with geometry matching the immobilization collar and bearing alignment markings. The holder was translated to align its central axis with the beam path in the x–y plane, while z-axis alignment (beam direction) was achieved using the point-source measurement described above.

#### Final anatomical registration using CBCT

Before each mouse irradiation, a SARRP CBCT scan was acquired. The CBCT volume was then rigidly aligned to the previously determined holder/isocenter coordinates, based on the known mechanical geometry of the holder, visible in the CBCT image. In all pre-treatment SARRP scans, identifiable 3D features of the bed were visible, enabling a visual alignment of the reconstructed images with the expected position defined above. Through this pipeline, each mouse anatomical position - acquired in the SARRP vertical configuration in the same holder as used for irradiation - was consistently mapped into the PET imaging coordinate system for the online monitoring.

### Tumor growth

Visible and palpable tumors were measured with a caliper twice per week up to a month post irradiation. To calculate the tumor volume V, the following formula was applied:

$${{{\rm{V}}}}=\frac{4}{3}{{{\rm{\pi }}}}{{{\rm{abc}}}}$$

where the tumor shape is assumed to be ellipsoidal, a and b are the length and width, and c the depth, calculated as the average of a and b.

### Toxicity assays

#### Skin toxicity scoring

Adapted from the Gesellschaft für Versuchstierkunde (GV-SOLAS) guidelines, the scoring system for the skin toxicity is divided in five grades: 0, no effect; 1, mild inflammation and dry skin, or white fur regrowth post irradiation; 2, moderate inflammation and desquamation; 3, acute inflammation and closed wound/crusts; 4, open wound failing to heal or necrosis. The termination criteria were reached at grade 4, and necrosis was never observed. To treat a starting inflammation, a topical treatment was applied locally (Bepanthen®, Bayer), which successfully healed animals showing up to grade 3 post irradiation.

#### Grip test

The grip test was used to assess the animal forelimb strength and was performed for the animals belonging to the tumor-free sham control, R and L groups every 2 weeks after the irradiation, after being trained with refined handling techniques to maximize cooperation during the task and optimize the data collection. The device used was the Grip Strength Meter-47200 (Ugo Basile®) equipped with a T-bar, where the animals were let grasping, and then pulled back until the grasp was released and the grip strength measured in Newton. In every test, a total of three measurements in which the animals were successfully grasping the bar with both paws were collected for each mouse.

#### Esophagus toxicity scoring

Animal weights were recorded weekly as a part of a regular follow-up. In the current study, a decrease in weight loss can be caused by the irradiation stress and additionally by the esophageal damage. For two weeks after irradiation, to prevent stress-induced weight loss the mice were fed DietGel® Boost food (ClearH_2_O, Westbrook, Maine, USA). To account for potential changes in mouse well-being induced by the esophagus toxicity, we have adapted the following scoring system from the GV-SOLAS guidelines: grade 0, no weight loss compared to the previous week, breathing undisturbed; 1, 10% weight loss; breathing undisturbed; 2, 15% weight loss, accelerated breathing; 3, 20% weight loss, marked breathing difficulties. Supportive care (e.g., boost food) was provided once grade 1 symptoms appeared, while animals were euthanized upon reaching grade 3.

#### Ex vivo histological analyses

To score the radiation-induced side effects on the healthy tissues, we performed several histological analyses. Animals were sacrificed according to the ethical protocol via intra-cardiac perfusion with 4% heparin sodium salt (Sigma-Aldrich, Missouri, USA) dissolved in PBS buffer, followed by 4% formaldehyde (ROTI ®Histofix, Carl Roth, Germany) for fixation. The organs of interest were dissected, left in 4% formaldehyde for 24 h and then transferred to EtOH 70% as a pre-step before dehydration. The full dehydration process was performed using the HistoCore Pearl dehydration station (Leica Biosystems, Germany) following the manufacturer’s protocol. After dehydration, the samples were embedded in ParaPlast Plus (Leica, Germany) and positioned in histological cassettes for cutting. The samples were sectioned in 5 µm thick slices with a microtome (Leica RM2235, Germany) equipped with C35 Type blades (Feather, Japan) and mounted using the floating method on glass slides (Labsolute, Germany). After overnight drying, the slides were stained with different staining protocols, according to the sample type.

#### Hematoxylin-eosin staining

Tissue staining was used to perform morphological analyses on all the samples, which were first deparaffinized in xylol, rehydrated trough ascending gradients of ethanol (100%, 96%, 70%), then rinsed in water, stained for 3 s in hematoxylin (Hematoxylin Gill no. 3, Sigma-Aldrich, Germany) and rinsed under tap water for 2 min. After rinsing, they were stained with eosin (Eosin-G 0,5% solution, Roth, Germany) for 2 min, washed three times in ethanol 80% to remove the excessive eosin, dehydrated again in ascending ethanol solutions (96%, 100%) and cleared in xylol. The slides were mounted with Eukitt and let dry overnight prior to acquisition.

#### Immunohistochemical staining

The tissue sections were deparaffinized and rehydrated as described above. The Akoya staining kit was used through the procedure, starting by the antigen retrieval step using an antigen retrieval chamber (DCARC0001, Biocare Medical) at 95 °C with AR9 buffer (Akoya 10xAR9, pH = 9, Akoya, Marlborough, MA). The slides were then cooled down and the samples circled with a hydrophobic marker before blocking with 0.3% H₂O₂ for 15 min. After 3 × 3 min washing with TBST, the slides were treated with blocking buffer for 15 min, washed once in TBST and then incubated with Ki-67 antibody (Rabbit mAb, d385, Cell Signalling Technology, Inc.) at 1:400 dilution for 1 hour. Afterwards, the slides were washed 3 × 3 min with TBST and then incubated with HRP-conjugated secondary antibody for 1 h. At the end of the incubation, the slides were washed as before and stained with DAB chromogen (BD Biosciences, San Diego, CA) for 5 min, then rinsed in tap water, counterstained with hematoxylin for 3 seconds and rinsed in tap water for other 2 min. The resulting slides were coverslipped with Aquatex® (Sigma-Aldrich, Germany) and let dry before acquisition.

#### Image acquisition and scoring

Akoya PhenoImager Fusion microscope (Akoya Biosciences, Fusion software 2.3.1, Marlborough, Massachusetts, USA), and the QuPath software (version 0.5.1), were used to quantify epithelial thickness, general morphology and the presence of Ki-67 positive cells in the immunohistochemical analyses. Esophageal staining were scored by using a simplified version of the EoE Histologic Scoring System (EoEHSS)^48^, using the following parameters: basal zone hyperplasia (BZH), surface epithelial alteration (SEA), and correlation of the hyperplasia areas with the presence of Ki-67-expressing cells. The degree of epithelial and lamina propria thickening was quantified from the hematoxylin-stained samples in QuPath. For each scanned segment, the epithelial thickness was measured as the distance from the keratin layer to the epithelial basal cell layer at regularly spaced intervals (approximately 60–80 µm), covering the entire circumference of the esophageal segment. Multiple measurements obtained from the same mouse were pooled, and the resulting distributions were used to generate histogram-based probability density curves for each animal, as well as group-average density curves.

### Statistics and reproducibility

To determine the sample sizes in the animal irradiation experiments, we chose sample sizes to obtain an expected effect size (Cohen´s *d*) of *d* = 1 using the online G*Power software (version 3.1.9.7). Animals were randomly allocated in the experimental groups, and the investigators were blinded to the groups during all the animal experiments. Mice from different treatment groups were housed in mixed cages whenever compatible with the experimental design, while control animals were housed separately. Individual animals were identified by ear marks and corresponding IDs recorded on cage cards and in the electronic database PyRAT to ensure consistent tracking throughout the study. The order of irradiation and imaging procedures was not considered a relevant source of confounding for the evaluated endpoints.

All the details regarding the individual statistical tests are specified in the respective figure captions. Data distribution was assumed to be normal where applicable. GraphPad Prism (software version 10.6.1, GraphPad Software, Boston, Massachusetts USA) was used for the statistical analyses of tumor growth, weights distribution, and grip test, which were analyzed with a 2-way ANOVA between curves. To quantify the differences in the esophageal thickness measurements, we used a non-parametric permutation test comparing the full distribution of per-mouse normalized probability density curves (performed in Python 3.11 using the itertools library). For each pair of groups, the effect size was defined as the integral of the absolute difference between group mean density functions, and statistical significance was determined from 10000 random permutations of mouse-to-group labels. Group medians were compared using Mann–Whitney tests applied to per-mouse median esophagus thicknesses.

## Results

### PET activity

Supplementary video 1 shows the PET activity distribution acquired during irradiation with a collimated, monoenergetic ^11^C beam reaching a dead mouse body, overlayed to the pre-irradiation CBCT scan. For all three irradiation modalities, the position of the activity peak was expected to be located at the 80% distal dose fall-off position^35^. This value was used to confirm the correct delivery during all the in vivo experiments, and it is marked with a white cross in the video. With respect to the isocenter (0 mm), the PET activity S peak was found to be at -4 mm (inside the compensator), the R peak at +4 mm (consistent with the 6.90 mm WET from the range shifter absorbers plus 1.16 mm WET residual in the plastic compensator), and the L one at +6 mm (corresponding to the 2 mm WET of the adsorbers in the range shifter).

The video demonstrates that we could move the beam quickly in any desired position, from the edge of the tumor down to the esophagus of the mouse and correctly guide the treatment via the in-beam PET imaging. Any millimetric deviation in the activity peak position is clearly visible, allowing for fast range verification and adaptation. The frame is indeed not disturbed by the considerable noise of the target activation, as unavoidable with stable beams. The video shows the dynamic PET signal integrated every 120 s. Notably, range could be accurately detected already after the first 25–30 s of PET acquisition, which, under our experimental conditions, would correspond to approximately 3·10^7^ particles delivering 0.4–0.5 Gy. Considering the estimated transmission efficiency of 1% through the beamline and with the probing collimator, only approximately 3·10^5^ particles effectively contributed to the in-beam PET signal for the range verification. Supplementary Fig. 4 shows the evolution of the activity peak position across different irradiation time intervals for the three irradiation modes: S, R and L in red, green and blue, respectively. The plotted points represent the mean activity peak position, while the error bars correspond to one standard deviation of the measured distributions within each interval. The dashed horizontal lines indicate the expected activity peak positions for each irradiation configuration. For each irradiation mode, the PET activity peak position is reported for the first 25 s of irradiation (“25 s Beam on”), during the full irradiation (“5 min irr. Beam on”), during the 10 minutes after the irradiation ends (“Decay Beam off”), during the 25 s immediately after modification of the range shifter configuration (“New c. Beam on”). The results demonstrate that the measured activity peak positions remain in good agreement with the expected beam ranges throughout the experiment, with deviations generally limited to approximately ±0.2 mm, corresponding to half of the voxel size. A clear transition in the activity peak position is observed during the first 25 s after changing the range shifter configuration for the new irradiation mode (“New c. beam on” in the scatter plot), during which the PET activity distribution does not yet fully reflect the new 80% dose fall-off position. This initial bias arises because the in-beam PET signal is still dominated by residual activity from the previous irradiation configuration, combined with the finite statistical buildup of newly induced activity. After this initial transient period the PET signal stabilizes, and the activity peak converges to the new position consistent with the updated range shifter setting. Supplementary Fig. 5A shows the total number of counts registered by the in-beam PET system every 25 s. A clear buildup of the signal is observed during beam-on irradiation, as well as the exponential decrease during the decay phase.

**Fig. 5:**
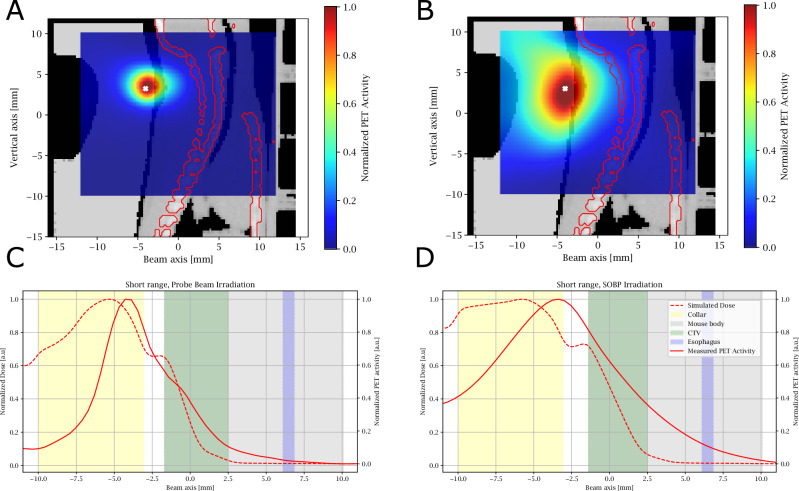
Irradiation in the “Short” position. **A** Online SIRMIO PET image of the positron activity distribution acquired during the ^11^C-irradiation in the S position with the collimated monoenergetic pristine beam overlaid on the SARRP scan of the mice under irradiation. **B** Online SIRMIO PET image acquired during the full SOBP irradiation with 20 Gy in the S position. Figure 1B shows the corresponding simulated dose distribution. **C**, **D** 1D profiles along the beam direction (z-axis) of the simulated dose (normalized to the target dose; dashed red line) and the measured (solid red) PET activity profiles, normalized to their maximum and laterally integrated on the BEV aperture (±1 mm x, 2–3 mm y) for the monoenergetic and SOBP configuration, respectively. The CTV, esophagus and mouse body are highlighted by green, blue and gray bands, respectively, while the compensator is depicted in yellow. The white “×” marker indicates the position along the beam axis corresponding to the 80% fall-off of the collimated ^11^C pure beam distribution.

The first irradiation position defined by the monoenergetic beam, S, is shown in Fig. 5A. The white “×” marker indicates the position along the beam axis corresponding to the 80% fall-off of the planned dose distribution. The full 20 Gy treatment image is shown in Fig. 5B, where the 80% fall-off position of the SOBP is also indicated. A Monte Carlo simulation was performed with FLUKA (see Methods for details) including the full experimental setup, the beam model and the PET signal formation in the detector, to account for the imaging process through the same reconstruction as for measured data. In Fig. 5C we show the simulated dose and the measured PET activity profiles along the beam direction, integrated over the beam’s-eye-view (BEV) aperture ( ± 1 mm) in the x–y plane transverse to the beam direction. Figure 5D shows the same profiles as Fig. 5C for the SOBP. In both Fig. 5C, D, the peak of the measured activity clearly coincides with the depth position of the simulated dose 80% fall-off, confirming the correct dose delivery. Supplementary video 2 shows the dynamic activity accumulation of the pristine Bragg peak and the SOBP in the S position. Supplementary Fig. 5B provide the same but in terms of the total number of counts registered by the in-beam PET system every 120 s, highlighting the build-up and decay phase for both the probe and SOBP irradiation.

**Fig. 6:**
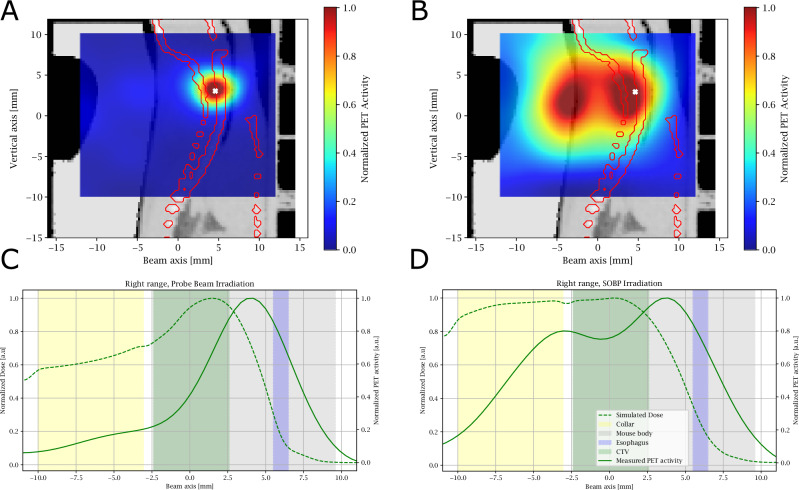
Irradiation in the “Right” position. **A** Online SIRMIO PET image of the positron activity distribution acquired during the ^11^C-irradiation in the R (Goldilocks range) position with the collimated monoenergetic pristine beam overlaid on the SARRP scan of the mice under irradiation. **B** Online SIRMIO PET image acquired during the full SOBP irradiation with 20 Gy in the R position. Figure 1C shows the corresponding simulated dose distribution. **C**, **D** 1D profiles along the beam direction (z-axis) of the simulated dose (normalized to the target dose; dashed green) and the measured (solid green) PET activity profiles, normalized to their maximum and laterally integrated on the BEV aperture (±1 mm x, 2–3 mm y) for the monoenergetic and SOBP configuration, respectively. The CTV, esophagus and mouse body are highlighted by green, blue and gray bands, respectively, while the compensator is depicted in yellow. The white “×” marker indicates the position along the beam axis corresponding to the 80% fall-off of the collimated ^11^C pure beam distribution.

**Fig. 7:**
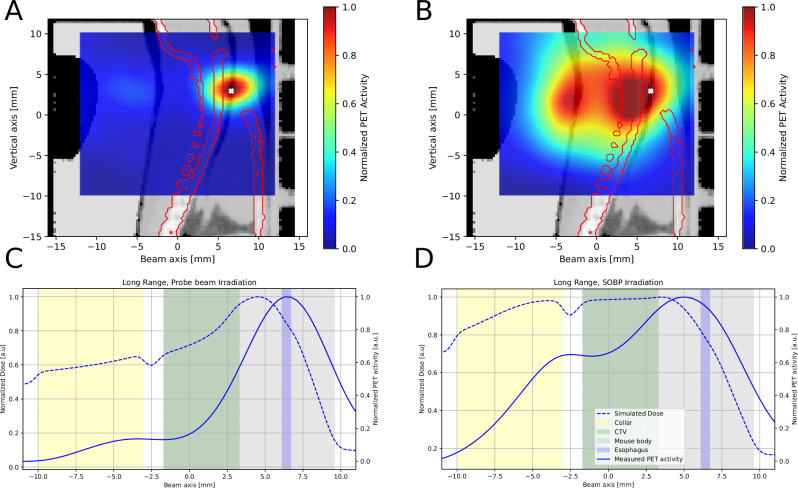
Irradiation in the “Long” position. **A** Online SIRMIO PET image of the positron activity distribution acquired during the ^11^C-irradiation in the L position with the collimated monoenergetic pristine beam overlaid on the SARRP scan of the mice under irradiation. **B** Online SIRMIO PET image acquired during the full SOBP irradiation with 20 Gy in the S position. Figure 1D shows the correspondent simulated dose profile. **C**, **D** 1D profiles along the beam direction (z-axis) of the simulated dose (normalized to the target dose; dashed blue) and the measured (solid blue) PET activity profiles, normalized to their maximum and laterally integrated on the BEV aperture (±1 mm x, 2–3 mm y) for the monoenergetic and SOBP configuration, respectively. The CTV, esophagus and mouse body are highlighted by green, blue and gray bands, respectively, while the compensator is depicted in yellow. The white “×” marker indicates the position along the beam axis corresponding to the 80% fall-off of the collimated ^11^C pure beam distribution.

Figure 6 shows the irradiation of a mouse in the R position, our Goldilocks range. In this case, the activity peak is moved to a position corresponding to the 80% dose fall-off covering the full CTV. In Fig. 7 we finally show the image and profiles for the L position, where the beam range is longer than the tumor size, and the ^11^C-beam stops after the spinal cord.

Supplementary videos 3 and 4 show the dynamic activity accumulation of the pristine Bragg peak and the SOBP in the R and L positions, respectively and Suppl. Fig. 5C, D report the corresponding temporal evolution in terms of total counts recorded by the in-beam PET system, integrated every 120 s. In both videos, a progressive increase in the activity signal is observed in the distal region of the compensator, close to the mouse skin. After the whole treatment ends ( ~ 1900 s), the activity within the mouse body decreases more rapidly than that within the compensator. This accelerated decline is attributable to the combined effects of radioactive decay and biological washout in the mouse tissue, while the compensator is subject only to physical decay. To quantify this behavior, the CBCT scan was resampled to the same coordinate system and spatial resolution as the PET images, and HU-based thresholding was applied to segment the volumes corresponding to the compensator and the mouse body. This approach enabled the evaluation of the total activity over time for each volume, separately. In Suppl. Fig. 6, the temporal evolution of the post-irradiation activity ratio between the mouse body and the compensator volumes is reported. The exponential fit exhibits a trend consistent with that reported in our previous study^35^ for 20 Gy SOBP irradiation. Compared to these earlier results, the enhanced detector sensitivity and optimized energy windows substantially improved image quality and quantitative accuracy, enabling a more detailed characterization of biological washout dynamics.

### Tumor control

**Fig. 8:**
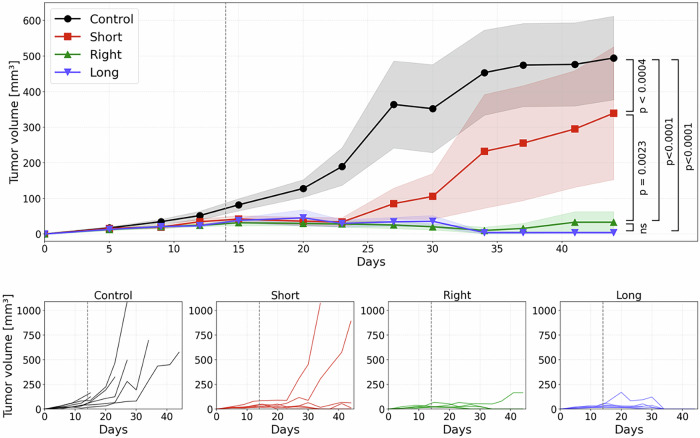
Tumor growth curves. The upper panel shows the average tumour volumes calculated from caliper measurements taken twice a week starting from the injection day (see Methods for details). The vertical grey line indicates the irradiation day. Individual tumour growth curves for the animals of the different treatment groups are shown in the lower panels. The groups are: 0 Gy tumour-bearing mice (black circle symbols, *n* = 7 animals), 20 Gy Short (S) range group (red square symbols, *n* = 6), 20 Gy Right (R) range group (green upward triangle symbols, *n* = 5), and 20 Gy Long (L) range group (blue downward triangle symbols, *n* = 6). Shaded areas of respective colors represent the regions of the standard errors of the mean for every group. During the observation period of 28 days, the animals reaching the permitted burden had to be sacrificed according to the ethical protocol; in such cases, the last measured tumor size was kept for subsequent calculations of the group-averaged tumor sizes. Growth trends were compared via the two-way ANOVA statistical test, and resulting *p*-values are displayed next to the respective bars.

Tumor growth curves are shown in Fig. 8. The osteosarcoma grows rapidly in the controls, with an average volume around 0.4 cm^3^ after one month from inoculation. The S group experiences a growth delay of about 10 days after the irradiation, but afterwards the average growth rate is similar to the control. These results reflect the lack of full tumor coverage shown in Fig. 5. On the other hand, tumors were completely controlled in all animals belonging to the groups R and L. The results are, therefore, an exact biological mirror of the PET images in Figs. 6, 7. By adapting the beam range we could get the desired tumor treatment results, providing evidence of real-time image guidance.

### Toxicity

Skin toxicity scoring in the tumor-bearing controls was complicated by the growth of the tumor that caused superficial lesions. In animals from the R and L groups, all of them bearing small tumors post-irradiation, skin toxicity was radiation-induced as shown in Suppl. Fig. 7. The erythema was successfully managed by application of a topical treatment. No irradiated animals showed skin toxicity grade > 3.

Except the mild skin toxicity, we expected no healthy tissue complications in the animals of R and S groups in case of correct image-guidance. Instead, we expected to see signs of damage to the esophagus and spinal cord for the L group.

One out of 9 animals in the L group showed signs of acute radiation-induced esophagitis 2 weeks after irradiation (Suppl. Fig. 8). This grade 3 morbidity is characterized by 20% weight loss, marked breathing difficulties, and hunched posture likely caused by pain. The µCT scan performed prior to sacrifice showed empty gastrointestinal tract, indicating an impaired food intake. Histological analysis of the esophageal tissues revealed a marked increase in the basal cells layer proliferation (hyperplasia), and surface epithelial alteration characterized by partial loss of the keratinized layer and organizational disruption.

**Fig. 9:**
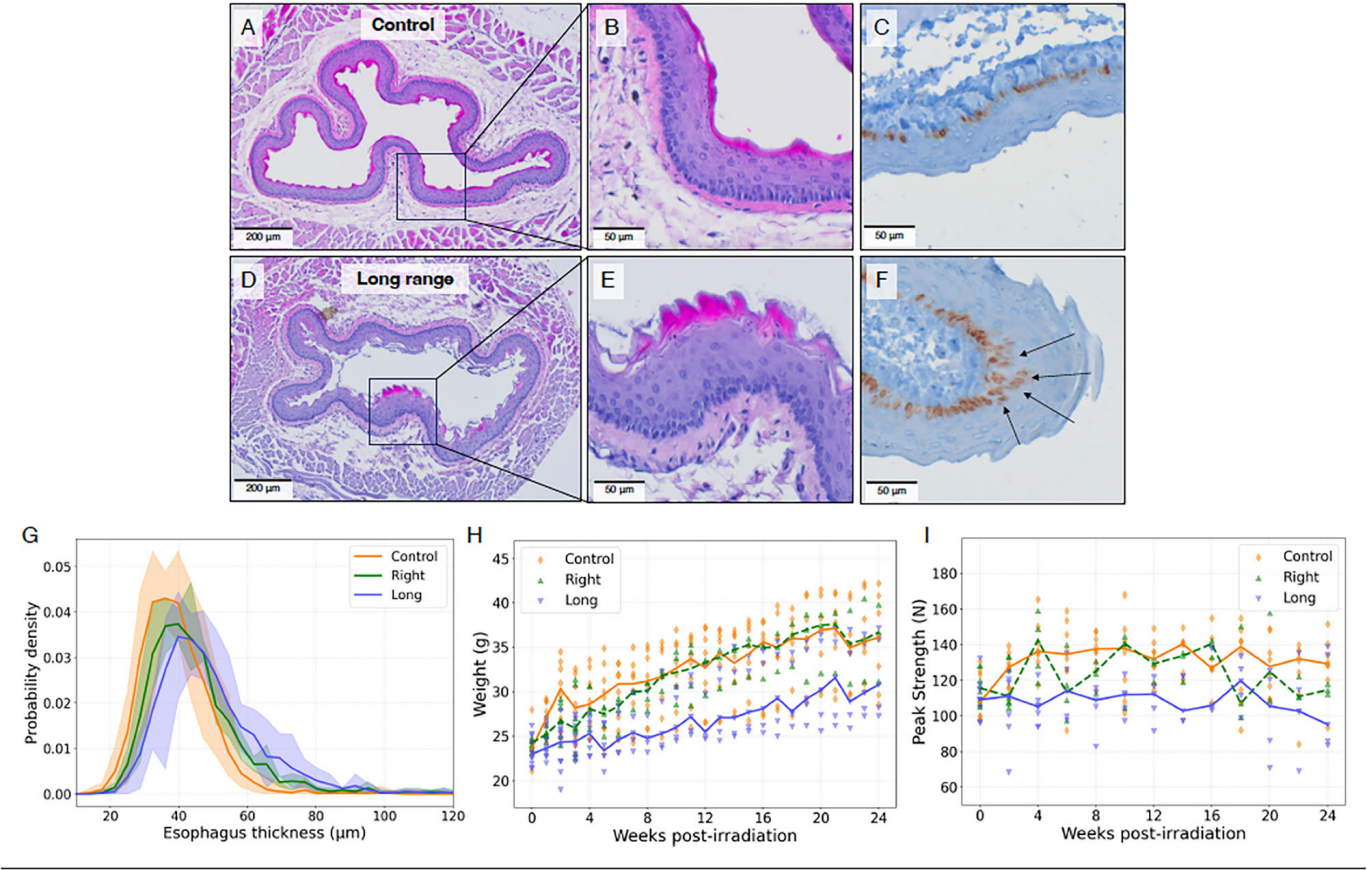
Normal tissue complications in irradiated mice. Representative pictures of hematoxylin-eosin staining (**A**, **B**, **D**, **E**) and Ki-67 immunohistochemistry (**C**, **F**) of the esophagus of the sham irradiated tumor-free controls (**A–C**) and animals treated in the L (long range) group (**D–F**). Samples from the L group are characterized by local areas of active cell proliferation in the basal layer (basal zone hyperplasia, BZH, with proliferating cells indicated by arrows in (**F**), which explains the increased thickness of the epithelium (**D**). **G** Distribution of esophageal epithelial thickness across experimental groups. Normalized histogram-based probability density curves are shown for non-irradiated, R and L groups in orange, green, and blue, respectively. For each group, the solid line represents the mean probability density across all mice, and the shaded region indicates the minimum-maximum range spanned by individual animals. A two-sided non-parametric permutation test revealed a significant difference between control and L mice (*p* = 0.0032), but not between control and R (*p* = 0.0844) mice. **H** Median body weights for control, R, and L treatment groups, measured weekly after the irradiation, shown as solid orange, dashed green, and solid blue lines, respectively. Individual mouse weight measurements are overlaid as points of the same color (diamond, upward triangle and downward triangle symbols for control, R and L group animals, respectively). Two-way ANOVA test showed that body weight differed significantly between the L group and both R and control groups (*p* < 0.0001), while no significant difference was observed between control and R groups (*p* = 0.3911). **I** Distribution of the bi-weekly grip strength measurements of each animal along the six-month post-irradiation follow-up period. Lines represent group median values, while symbols represent individual animal measurements (tumour-free sham control group: solid orange line, diamond symbols, *N* = 8; R group: green dashed line, upward triangle symbols, *N* = 8; and L group: solid blue line, downward triangle symbols, *N* = 9). Individual values of grip strength were calculated as averages of three measurements taken for every mouse at every time point. Two-way ANOVA test showed that grip strength differed significantly between L group and both R and control groups (*p* < 0.0001), while no significant difference was observed between control and R groups (*p* = 0.0532). Because several mice in the R and L groups developed metastases during follow-up, the number of animals decreased over time, resulting in final group sizes of *N* = 3 (R) and *N* = 5 (L).

The remaining 8 animals in the group L reached the end of the planned post-irradiation follow-up period of 6 months. Figure 9A–F show representative histological sections of the esophagus from control (A–C) and L group (D–F) animals. Figures 9A, D display full transverse esophageal sections, illustrating overall tissue architecture. While the epithelium of control samples remained intact and regularly stratified (Fig. 9B), in the L group, areas of increased epithelial thickness were frequently observed (Fig. 9E). These regions of thickened epithelium coincided with an elevated density of Ki-67-positive proliferating cells (indicated by black arrows in Fig. 9F), while control tissues showed Ki-67-positive nuclei only in the basal cell layer, which is the normal condition in the esophagus. Figure 9G shows the group-average epithelial thickness distributions for the L, R, and control groups. The distribution of the L group is skewed toward larger thickness values, indicating that some irradiated areas of the esophagus exhibited abnormal epithelial thickening, arguably where the beam traversed the organ. A statistically significant increase in median value is observed in the L group compared to the control, whereas the R group does not exhibit a significant difference (Suppl. Fig. 9).

During the 6-months post-irradiation follow-up, mice were regularly weighted and subjected to the grip test to assess spinal cord toxicity. Mice in the L group gained substantially less weight than those in the control and R groups (Fig. 9H), further supporting the presence of radiation-induced esophageal damage.

For the myelopathy analysis, Fig. 9I presents the distribution of the grip strength values for each animal as a function of time after irradiation. A significant decrease in grip strength was observed the L group compared to both the sham and R groups, whereas the latter two do not differ significantly from each other.

Taken together, these findings demonstrate that the long range irradiation induced toxicity affecting both the spinal cord and the esophagus, while the right range irradiation caused no measurable biological impairment in either tissue.

## Discussion

Radioactive ion beams offer a unique opportunity for in-beam PET real-time range-guided particle therapy. Owing to their high counting rate and the strong correlation with the distal dose fall-off of the beam, they enable dynamical and fast range monitoring and adaptation during irradiation. In this study, we demonstrated an “aim-and-shoot” approach, in which a monoenergetic narrow beam acts as a pointer to localize the Goldilocks target within the animal, followed by the delivery of the therapeutic dose in the SOBP.

The anatomical image is taken shortly before exposure in the same treatment position, and this turned out to be essential to detect anatomical discrepancy with the planned ones (see Fig. 4). The beam can then be moved longitudinally in any position and monitored in real-time as visualized by PET, to identify the spot where the SOBP dose has to be delivered (Supplementary video 1, Supplementary Fig. 4 and 5A). The biological outcomes were consistent with expectations: when the range was too short (S; Fig. 5, Supplementary video 2), tumor control was not achieved (Fig. 8); when the range was too long (L; Fig. 7; Supplementary video 4), normal tissue toxicity was observed (Fig. 9; Supplementary Figs. 8, 9). In the Goldilocks position identified by the monoenergetic beam (R; Fig. 6; Supplementary video 3), we achieved tumor control without toxicity. The whole range spanned by the beam (S to L) in the mouse was around 10 mm, demonstrating an impressive resolution of 0.4 mm in vivo with radioactive ion beams.

We estimated that the position of the activity peak could reliably track the distal dose fall-off with sub-millimetric accuracy ( ± 0.2 mm uncertainty) within 25 s after delivering 0.4 Gy (see Supplementary Fig. 4). Such performance paves the way for in-beam PET–guided range-adaptation treatments that are not achievable with proton or stable carbon ion beams, where higher statistics and dose are typically required. Previous range-guided approaches in a human phantom with proton beams^38,49^ achieved, for comparable doses, PET-based range verification within approximately 60 s of acquisition with a reported uncertainty of about 0.5 mm. These results demonstrate that radioactive ion beams can enable rapid, low-dose, high-precision in vivo range verification, supporting their applicability for real-time adaptive particle therapy.

Limitations of the present work should be considered. First, this is a proof-of-principle study conducted in a murine osteosarcoma model with relatively simple and homogeneous anatomy compared to the heterogeneous tissues and complex geometries encountered in patients. This may impact the direct translatability of the observed PET–dose correlations and range verification and adaptation accuracy. Indeed, the “aim-and-shoot” workflow assumes accurate knowledge of the target WET and controlled beam delivery conditions. Residual uncertainties in WET estimation, positioning, and patient motion—particularly respiratory motion, negligible in our experimental setting but significant in patients—were not addressed here and could reduce accuracy in clinical applications. To date, the feasibility of in-beam PET monitoring and its sensitivity to anatomical variations in patients treated with radioactive ion beams has mainly been investigated through simulations in head-and-neck cancer^50^. Moreover, patients may exhibit more complex and spatially heterogeneous washout dynamics, potentially degrading the spatial correlation between PET activity and delivered dose over time.

Third, our work is limited to passive beam delivery systems. Clinical delivery conditions may further influence PET images, particularly for multiple treatment fields and Pencil Beam Scanning (PBS) over extended volumes, where multiple time-structured spot delivery modifies the spatiotemporal activity distribution. Nevertheless, reconstructed PET images represent the cumulative 3D activity distribution integrated over pre-defined time window rather than individual spot deliveries. We demonstrate the feasibility of performing accurate range verification and adaptation after 25 s from the start of the treatment^25,46^ irradiation by localizing the activity peak position along the beam axis that accurately reflects the 80% of the dose distal-fall off position in the target. However, in our irradiation settings, we observed that subsequent treatment adaptation can temporarily impair the correct localization of the dose fall-off position within the first 25 s after the plan/range shifter modification. As shown in Supplementary Fig. 4 (“New c. Beam on”), the PET activity peak during this initial interval does not yet correspond to the expected range of the new configuration due to the residual activity from the and decay of short-lived isotopes. After additional 25 s, the activity distribution stabilizes, and the peak position converges toward the expected beam range.

Finally, although radioactive ion beams offer clear advantages in terms of PET signal-to-noise-ratio and spatial correlation with dose, their production, handling, and clinical implementation remain technologically challenging and are currently limited to specialized facilities, which may constrain near-term scalability. Nevertheless, ongoing plans toward clinical implementation are currently being pursued at QST in Japan^51^, at CERN using ISOL-produced beams^52^, and at GSI employing the in-flight separation technique^53^.

Despite these limitations, this work represents the first demonstration of in-beam PET–real-time range-guided therapy using radioactive ion beams in an animal model, highlighting their strong potential toward future real-time adaptive strategies.

## Supplementary information

Transparent Peer Review file

Supplementary material

Description of Additional Supplementary Files

Supplementary video 1

Supplementary video 2

Supplementary video 3

Supplementary video 4

## Data Availability

All the relevant data is contained within the article and its Supporting Information files. Raw data are available on Fighsare from 10.6084/m9.figshare.31444906.
