# Supplementary material for: Radioactive ion beam range adaptation in mouse tumors using in-beam PET
Source: Commun Med (Lond). 2026 Jul 14;6:393. doi: 10.1038/s43856-026-01786-1 (PMC13369987; doi:10.1038/s43856-026-01786-1)

Supplementary Material

Supplementary Figure 1. **Water phantom measurements**. Water phantom measurements of the **A.** vertical and **B.** horizontal 2D dose distributions in the central plane along the beam direction respectively. **C.** Water phantom measurement of the beam spot 2D dose distribution at the minimal water equivalent depth (z = 21 mm) inside the phantom. **D.** Horizontal and vertical beam profiles acquired on the central line of the beam spot shown in panel C. All the data are normalized to the maximum dose.

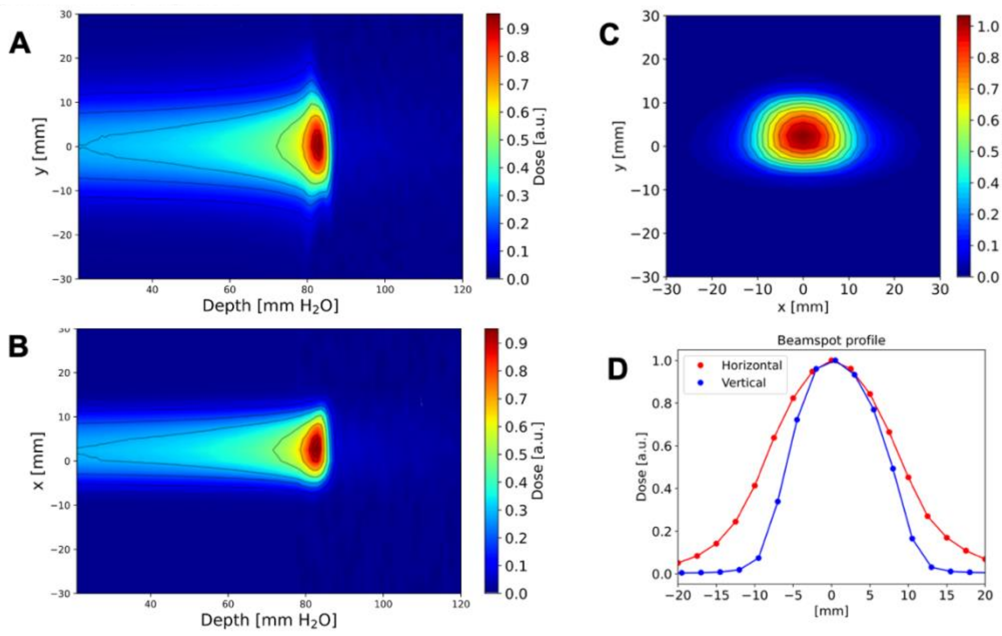

Supplementary Figure 2. **Dose profiles experimental setup.** Schematic representation of the experimental setup used to measure 1D depth dose profiles at the mouse irradiation position shown in Figure 3B (black line). The PEAKFINDER was placed with the front window at the isocenter position. Before reaching the PEAKFINDER window the beam was passing the 2DRM, the collimators and the static absorber component (7.0 mm WET polyethylene and 51.5 mm WET aluminum). Created in BioRender. Boscolo, D. (2026) https://BioRender.com/4fipeqb.

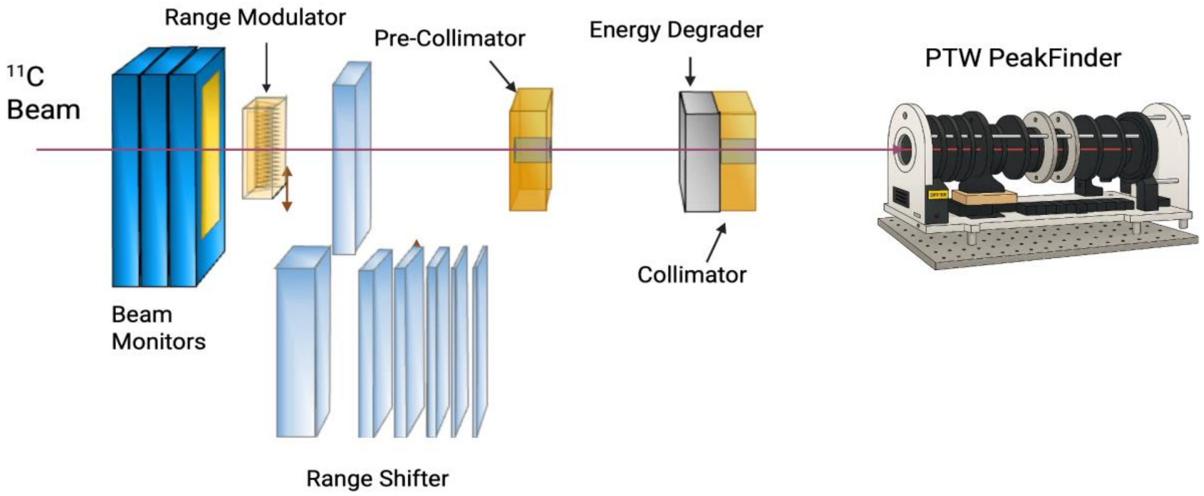

Supplementary Figure 3. **SOBP dose maps experimental setup**. Schematic representation of the experimental setup used to measure 1D and 2D SOBP dose maps at the mouse irradiation position, with all the absorbers and collimators placed and aligned as for the R range mouse irradiations (15 mm WET polyethylene and 51.5 mm WET aluminum). Measurements are shown in Figure 3B (Blue dots) and 3C. The PTW OCTAVIOUS 1600 XDR detector was placed free in air with the detector window at the isocenter position. 2D dose maps at different depths in water were measured by increasingly adding PMMA slabs of different thickness in front of the detector. Created in BioRender. Boscolo, D. (2026) https://BioRender.com/4fipeqb.

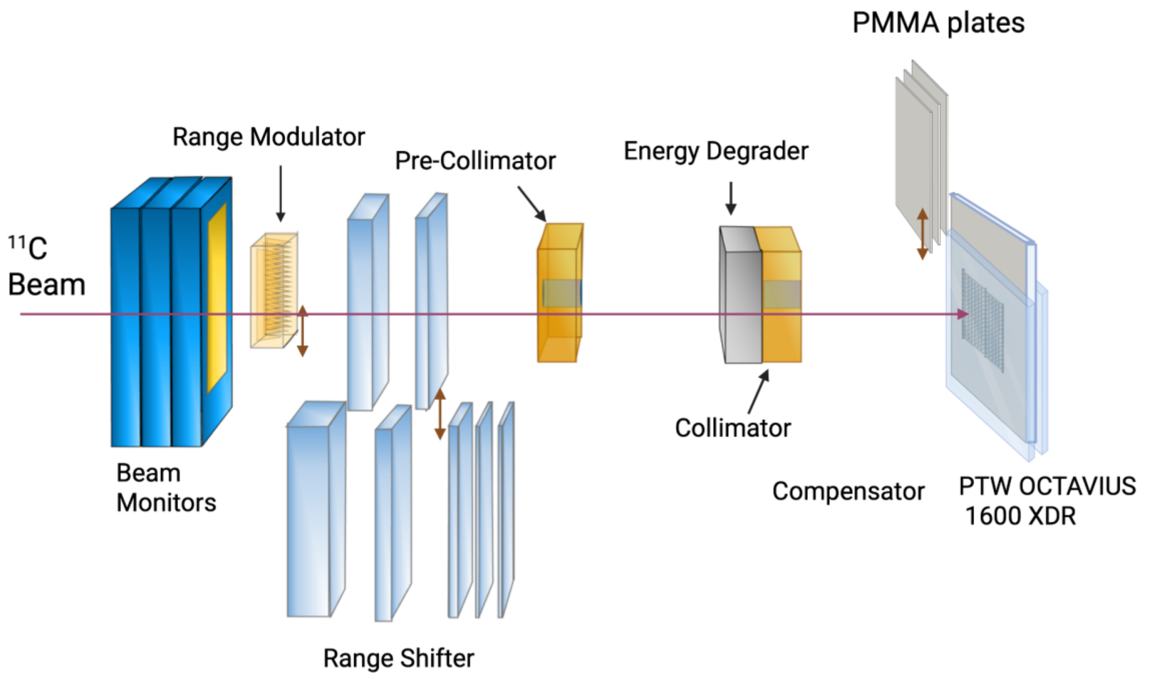

Supplementary Figure 4. Peak position at different acquisition times. Activity peak position measured across different irradiation time (see Supplementary Figure 5A) intervals for the S, R and L irradiation configurations, respectively. The mean activity peak position and one standard deviation are reported for measurements acquired during the first 25 s of irradiation ("25 s Beam on"), during irradiation ("5 min irr. Beam on"), during the 10 minutes after irradiation ends ("Decay Beam off"), and immediately after modification of the irradiation configuration ("New c. Beam on"). Dashed horizontal lines indicate the expected activity peak positions for each irradiation configuration (-4 mm, 4 mm, and 6 mm, respectively).

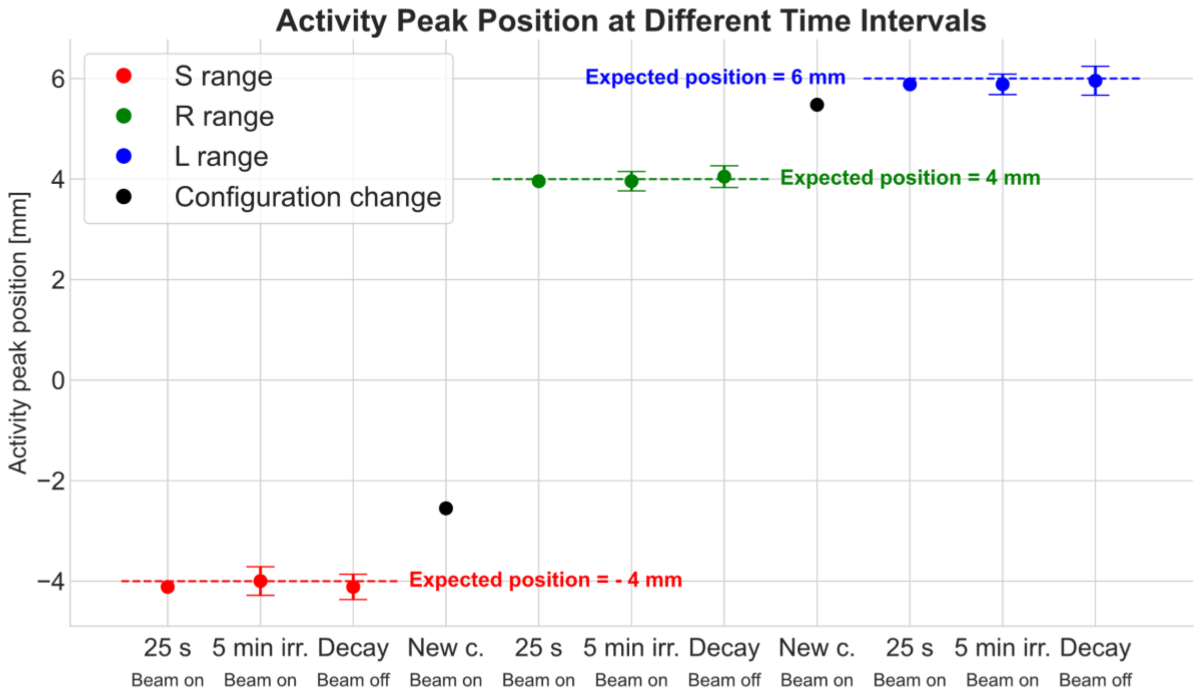

Supplementary Figure 5. **Online PET counts**. Temporal evolution of in-beam PET counts during irradiation (vertical bands) and after beam delivery. Panel **A** shows the number of counts recorded by the in-beam PET system as a function of time, integrated every 25 s intervals during the probing-beam protocol used to characterize the three irradiation modes (S, R, and L), see Supplementary video 1. Each mode consists of a 5 min irradiation period, with vertical bands highlighting each range 5 min of irradiation, followed by a 10 min beam-off decay phase (see Supplementary Figure 4), after which the range shifter configuration is modified to switch to the next irradiation condition. Panels **B–D** report the corresponding signal evolution for the S, R, and L irradiation configurations, respectively, including the transitions between probing beam (5 min) and SOBP (20 min) irradiation conditions induced by changes in the range shifter settings and the introduction of the 2D Range Modulator for SOBP. The data illustrate the consistent build-up of PET activity during beam-on irradiation and its subsequent decay after irradiation ends.

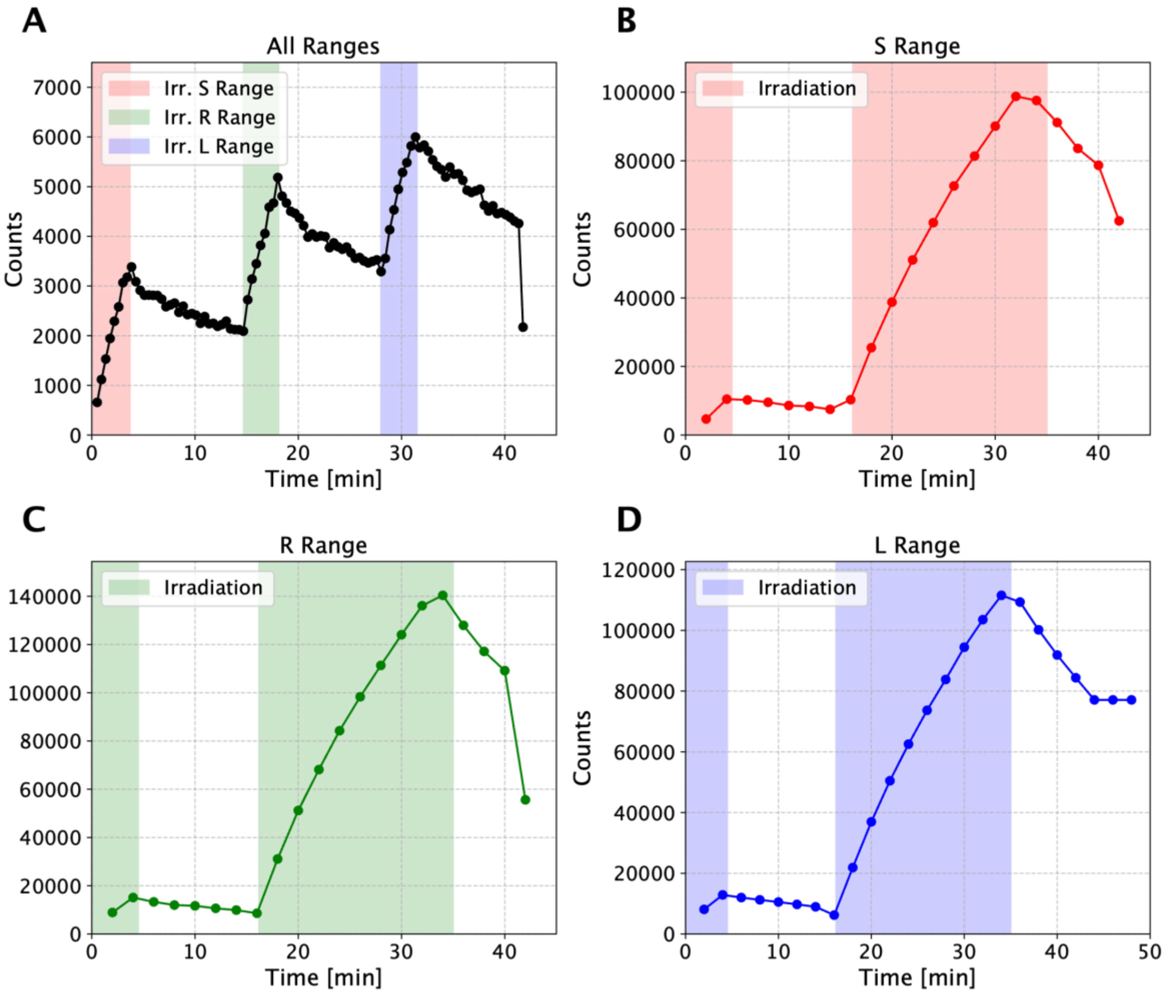

Supplementary Figure 6. Washout. Individual post-irradiation ratios of the activity measured in the mouse body to that in the compensator, acquired after 20 Gy SOBP exposure for the mice presented in Figures 6 and 7. The fitted function follows the model reported in ref.[35] and is shown as a dashed black line. The blue band represents the range of fitted biological decay constants obtained separately for the two datasets (long- and right-range acquisitions).

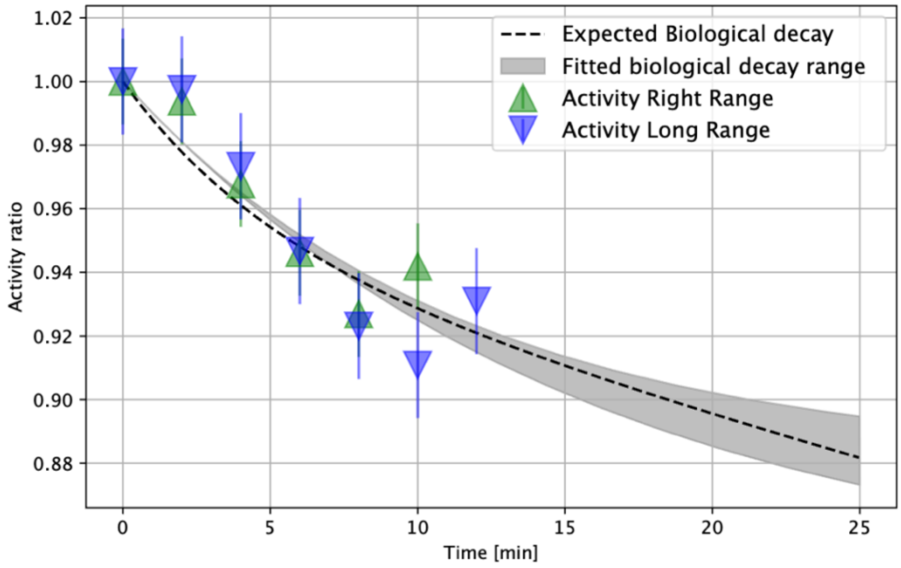

Supplementary Figure 7. Skin toxicity in tumour-bearing mice. Bars indicate the percentage of animals in each group exhibiting skin toxicity grades 0–4. Groups include non-irradiated tumor-bearing controls, tumor-free sham controls, as well as short (S), right (R) and long (L) range groups. Grade 4, which required euthanasia of the animals, was observed only in the tumour-bearing control group and was caused by the development of lesions on the tumour site. For each experimental group, animal numbers are detailed in the figure.

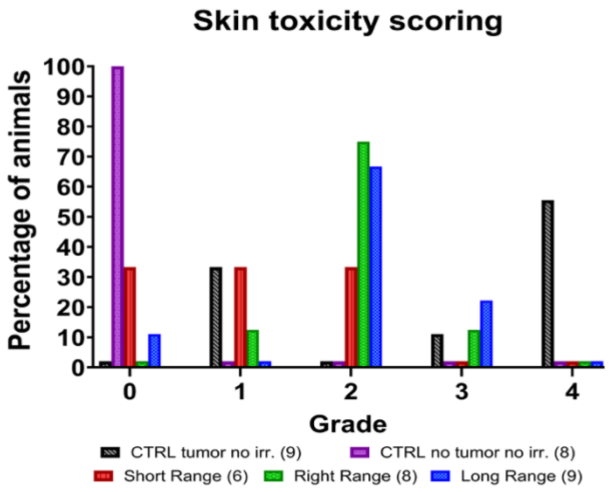

Toxicity Grade	Manifestation
0	No effect, or healed post irradiation
1	Mild inflammation and dry skin, or white fur regrowth post irradiation
2	Moderate inflammation, desquamation
3	Acute inflammation, closed wound/crusts
4	Open wound failing to heal (termination of the animal required)

Supplementary Figure 8. Acute esophageal toxicity. Panels **A-B** show the photo and the μCT scan of a mouse from the L group two weeks after irradiation, manifesting breathing issues, severe weight loss, and altered food intake, which was indicated by the hunched posture and air-filled areas of the gastrointestinal tract seen on the CT. The animal had to be euthanized two weeks post irradiation according to the ethical protocol, having reached Grade 3 toxicity. The hematoxylin-eosin staining of the esophagus (**C**) of a sham irradiated control mouse is compared to esophagus (**D**) of the L-group animal shown in the panels **A-B**. Circles indicate the difference between the normal keratinized epithelium of the esophagus (**E**) and the surface epithelial alteration (SEA, **F**), with the partial loss of the keratinised layer. The arrows show the normal monolayer of basal cells (**E**) and the basal zone hyperplasia (BZH, **F**). The curly brackets show the normal epithelium thickness (**E**) and the consequence of the hyperproliferation of the basal cells layer, resulting in a thickened epithelium (**F**). The different parts of the esophagus are described in the panel and the figures **E-F** are the magnifications of the **C-D**, respectively.

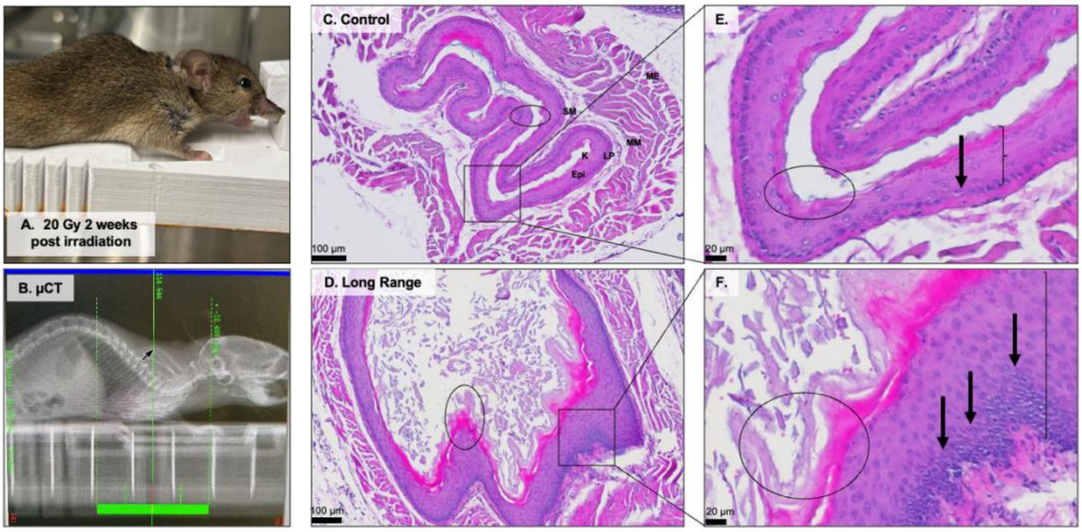

K: keratinised epithelium; Epi: epithelium; LP: lamina propria; SM: submucosa; MM: muscolaris mucosa; ME: muscolaris externa

Supplementary Figure 9. Esophageal epithelium thickness. Distribution of per-mouse median esophageal epithelial thicknesses, calculated from all individual measurements in the control (blue), R (green), and L (orange) irradiation groups. Frequency distributions of the esophagus thickness are shown in Fig. 9G. Boxes represent the interquartile range (25th–75th percentiles), central orange lines are group medians, and whiskers indicate the full measurement range (minimum to maximum). Individual points correspond to single animals. Pairwise group comparisons were performed using Mann–Whitney tests applied to per-mouse medians, and resulting p-values are shown above the corresponding brackets.

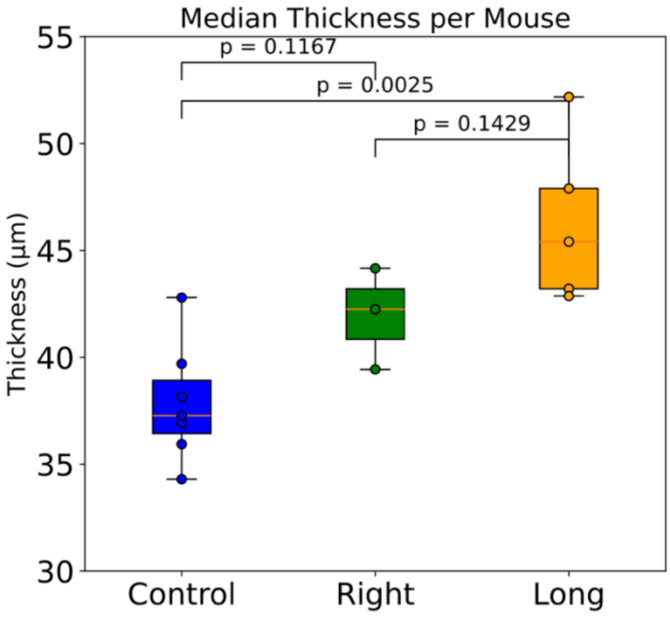

Supplement: Supplementary file 2 — Supplementary material [file 43856_2026_1786_MOESM2_ESM.pdf]
