# Supplementary material for: Radioactive ion beam range adaptation in mouse tumors using in-beam PET
Source: Commun Med (Lond). 2026 Jul 14;6:393. doi: 10.1038/s43856-026-01786-1 (PMC13369987; doi:10.1038/s43856-026-01786-1)
Supplement: Supplementary file 3 — Description of Additional Supplementary Files [file 43856_2026_1786_MOESM3_ESM.docx]

**Supplementary videos**

**Supplementary Video 1. Dynamic beam repositioning.** Dynamic PET activity signal acquired during three consecutive irradiations (corresponding to the three range settings) with a 5 Gy collimated monoenergetic ¹¹C beam, overlaid on the SARRP scan of the dead-mouse under irradiation. Each irradiation consists of 4 min of beam delivery followed by 10 min of decay. After each irradiation, range shifter plates were removed, allowing the beam to reach greater depths within the mouse body (from S to L range). Three distinct color scales are used to indicate the activity levels for each range, reflecting both isotope decay and cumulative signal build-up over time (see also **Supplementary Figure 5A**). Each frame corresponds to 2 min of PET acquisition. The white “×” markers indicate the positions along the beam axis corresponding to the 80% fall-off of the collimated ^11^C-beam distribution at the three different ranges in the mouse body.

**Supplementary Video 2**. **S-experiment imaging**. Dynamic PET activity signal accumulation during two consecutive irradiations for the S range setting, one with the 5 Gy collimated monoenergetic ¹¹C beam and the following one with the 20 Gy SOBP, overlaid on the SARRP scan of the alive mouse under irradiation. Monoenergetic irradiation consists of 4 min of beam delivery followed by 10 min of decay, while for the SOBP 20 min of beam delivery and 10 min of decay. After the monoenergetic irradiation, the range modulator was added and range plates were removed, allowing the beam to reach the same depth obtained with the monoenergetic probe beam used for range verification. Two distinct color scales are used to indicate the activity levels for each irradiation, reflecting both isotope decay and cumulative signal build-up over time (see also **Supplementary Figure 5B**). Each frame corresponds to 2 min of PET acquisition. The white “×” marker indicates the position along the beam axis corresponding to the 80% fall-off of the collimated ^11^C pure beam distribution.

**Supplementary Video 3. R-experiment imaging**. Dynamic PET activity signal accumulation during two consecutive irradiations for the R range setting, one with the 5 Gy collimated monoenergetic ¹¹C beam and the following one with the 20 Gy SOBP, overlaid on the SARRP scan of the alive mouse under irradiation. Monoenergetic irradiation consists of 4 min of beam delivery followed by 10 min of decay, while for the SOBP 20 min of beam delivery and 10 min of decay. After the monoenergetic irradiation, the range modulator was added and range plates were removed, allowing the beam to reach the same depth obtained with the monoenergetic probe beam used for range verification. Two distinct color scales are used to indicate the activity levels for each irradiation, reflecting both isotope decay and cumulative signal build-up over time (see also **Supplementary Figure 5C**). Each frame corresponds to 2 min of PET acquisition. The white “×” marker indicates the position along the beam axis corresponding to the 80% fall-off of the collimated ^11^C pure beam distribution.

**Supplementary Video 4.**  **L-experiment imaging**. Dynamic PET activity signal accumulation during two consecutive irradiations for the L range setting, one with the 5 Gy collimated monoenergetic ¹¹C beam and the following one with the 20 Gy SOBP, overlaid on the SARRP scan of the alive mouse under irradiation. Monoenergetic irradiation consists of 4 min of beam delivery followed by 10 min of decay, while for the SOBP 20 min of beam delivery and 10 min of decay. After the monoenergetic irradiation, the range modulator was added and range plates were removed, allowing the beam to reach the same depth obtained with the monoenergetic probe beam used for range verification. Two distinct color scales are used to indicate the activity levels for each irradiation, reflecting both isotope decay and cumulative signal build-up over time (see also **Supplementary Figure 5D**). Each frame corresponds to 2 min of PET acquisition. The white “×” marker indicates the position along the beam axis corresponding to the 80% fall-off of the collimated ^11^C pure beam distribution.
