# Supplementary material for: Radioactive ion beam range adaptation in mouse tumors using in-beam PET
Source: Commun Med (Lond). 2026 Jul 14;6:393. doi: 10.1038/s43856-026-01786-1 (PMC13369987; doi:10.1038/s43856-026-01786-1)
Supplement: Supplementary file 1 — Transparent Peer Review file [file 43856_2026_1786_MOESM1_ESM.pdf]

nature portfolio

Peer Review File

Radioactive ion beam range adaptation in mouse tumors using in-beam PET

Corresponding Author: Professor Marco Durante

This file contains all reviewer reports in order by version, followed by all author rebuttals in order by version.

Version 0:

Reviewer comments:

Reviewer #1

(Remarks to the Author)
The authors demonstrates the first result of application of PET-guided adaptive radiotherapy in an animal study. This can improve accuracy of dose delivery in particle therapy. The manuscript is well written and illustrated but a few issues should be addressed before publication.

Major comments:
1) The manuscript lacks any discussion of the limitations of the present study, the disadvantages of the proposed strategy, and the potential technical challenges associated with the clinical implementation.
2) Lines 164-165: how many spills and particles in total were delivered to the spot for the confirming the range? The dose of 5 Gy seems significant for probing beams. Can it be reduced and to what value?
3) Lines 313-315: how does the signal-to-noise ratio of a PET image relate to particles per spot? Do you have any quantitative data on this? I wonder what minimum beam intensity would allow for range verification. If it is not possible to significantly reduce the intensity of the probing beams, perhaps it can be possible to take them into account when calculating the dose during treatment planning? Please clarify these points in relation to the clinical implementation of your strategy.

Minor comments:
4) Line 143: maybe "0.4 mm" instead of "0.40 mm"
5) Figure 2: arrow for "Compensator" was not provided

Reviewer #2

(Remarks to the Author)
I would like to commend the authors for this innovative and technically demanding study. The demonstration of real-time in-beam PET for monitoring radioactive ion beam delivery in a pre-clinical mouse model is a valuable contribution to the field, and the methodology and statistical analyses are sound, comprehensive and well-executed.

My primary concern relates to the framing of the work. The manuscript implies in several places that this constitutes a demonstration of adaptive radiotherapy, with repeated reference to "real-time" adaptation. However, the study does not appear to include an actual modification of the treatment plan in response to the PET signal. Indeed, the manuscript itself states that the probe beam is used to confirm the range, rather than to determine or adapt the initially planned delivery. What is demonstrated is the feasibility of real-time in-beam PET as a monitoring tool, which is an important and meaningful achievement in its own right, but distinct from real-time adaptive delivery. I would encourage the authors to refocus the manuscript on the added value of PET guidance and its potential role as an enabler of adaptive workflows, rather than presenting the work as a demonstration of a complete adaptive solution. This reframing would, in my view, result in a more accurate and ultimately more compelling narrative.

Relatedly, the manuscript would benefit from a discussion of what would be required to bridge the gap toward genuine real-time adaptation, including how dose delivered early in a fraction would be accounted for when modifying the remaining plan-to-be-delivered, and what computational and dosimetric infrastructure this would demand. In this context, the probe beam dose of 5 Gy warrants particular attention. This is a substantial dose, and it would be valuable to discuss how this would be

managed in a scenario where the PET signal reveals an unexpected discrepancy rather than confirming the expected range. The authors might also consider whether 5 Gy represents the minimum dose required to achieve a usable signal, and whether the approach would remain viable in conventionally fractionated treatment schemes.

A further point concerns the delivery system itself. The current implementation is demonstrated on passive beam delivery system, whereas the majority of modern particle therapy facilities use active scanning systems. The authors should discuss the applicability of this approach to scanning-based delivery, and whether any transition back to passive systems would be required to implement such PET guidance based workflows in contemporary clinical settings.

The manuscript would also benefit from a more candid discussion of limitations, including the scalability of the approach to larger animals and humans, potential confounds on the PET signal such as biological washout, patient motion, and irradiation timing, and the translational relevance of the mouse model more broadly.

Minor comments:
L68–69: The statement that images do not provide information on dose deviations directly is technically correct, but it would be worth acknowledging that dose deviation estimates can nonetheless be derived or inferred from such images. A small rephrasing to reflect this nuance would be helpful.

L140: To improve readability, it would be beneficial to briefly indicate at this point what the two irradiation conditions are, rather than leaving this implicit until later.

L165: The manuscript describes the feedback as instantaneous; however, 25 seconds may not meet the threshold typically associated with that term in a clinical or real-time monitoring context.

L184–189: The suggestion that positional discrepancies necessarily call for online adaptation may be too strong. In conventional clinical practice, such discrepancies would typically first prompt a review of immobilization and positioning protocols, with online adaptation representing one possible but not necessarily the primary response. A more measured phrasing here would better reflect clinical reasoning.

Sincerely,
Arturs Meijers
Paul Scherrer Institute

Reviewer #3

(Remarks to the Author)
The manuscript presents a novel in vivo study investigating whether radioactive ion beams can support in-beam PET-guided adaptive particle therapy. Using an osteosarcoma C3H mouse model, the authors acquired CT images in the same vertical position used for irradiation and visualized a therapeutic β+-emitting ^11C beam in real time with the SIRMIO in-beam PET scanner. Three predefined beam depth settings were tested, corresponding to short, right, and long ranges, with the goal of identifying the “Goldilocks” beam position that adequately covers the tumor while minimizing dose to surrounding normal tissues. In addition to the imaging component, the study also evaluates tumor response and normal tissue toxicity across the different beam range conditions. The authors show that different beam positions generate spatially resolved PET signals associated with distinct biological outcomes, and they propose that radioactive ion beams may enable real-time adaptive treatment. Overall, the work is creative, technically interesting, and potentially important because it moves this concept into an in vivo setting and suggests a path toward PET-guided range assessment in particle therapy.

My main concern is that the manuscript may currently overstate the degree of “real-time adaptive therapy” demonstrated. Based on the described short/right/long beam design, the work appears to provide a strong in vivo proof-of-concept for PET-guided beam range assessment or range selection, but not yet a fully realized closed-loop adaptive radiotherapy workflow as many readers may interpret that term. The authors should therefore define much more clearly what was adapted, when the adaptation occurred, and whether the in-beam PET signal was used prospectively during the same experiment to alter treatment delivery. In addition, the “Goldilocks range” concept is appealing but needs a clearer quantitative decision criterion, including how the optimal range was determined from the PET signal and how reproducible that determination was across animals. The manuscript would also benefit from a more careful discussion of the known mismatch between PET activity and actual dose distribution, including whether and how the radioactive ion beam approach mitigates this limitation. Finally, some of the language, particularly the suggestion that radioactive ion beams may be the “ideal tool,” feels stronger than the current data can fully support and should be toned down unless justified with broader comparison and validation.

Specific comments,
1. Line 157, it was not clear how the three plans (S, R and L) were created. Is it based on a previous CT scan for the animal? And the compensator was generated through some planning process to match with the particular anatomy site you plan to treat?
2. Line 164, how long is the delivery for the first 5Gy spot? Is the PET concurrent with the delivery or after the delivery? How did you decide to choose 5Gy? It seems to be quite high dose that choose for the range calibration purpose.

3. Line 166, how did you confirm the range from the probe irradiation? Is there a particular location you're comparing with?
4. Line 176, does the subject stay at the same location between the CBCT image and in-beam PET?
5. Line 187, it's quite confusing that you claim adaptive workflow but the workflow cann't handle anatomy change between the uCT and delivery day CBCT.
6. Line 196, it would be better to have a quantitative comparison between the position of activity peak and the 80% distal fall off position. Also it is not clear how many animals were used in the experiment for a statistical analysis.
7. Line 300, for general adaptive purposes, beside the distal range information, one still need lateral profile info and spot intensity info. It's not clear how that information can be obtained with in-beam PET images.

Version 1:

Reviewer comments:

Reviewer #1

(Remarks to the Author)
The authors addressed the comments and improved the manuscript.

Reviewer #2

(Remarks to the Author)
I would like to thank the authors for the revised version of the manuscript. The points raised have been thoroughly clarified and addressed.

Open Access This Peer Review File is licensed under a Creative Commons Attribution 4.0 International License, which permits use, sharing, adaptation, distribution and reproduction in any medium or format, as long as you give appropriate credit to the original author(s) and the source, provide a link to the Creative Commons license, and indicate if changes were made.
In cases where reviewers are anonymous, credit should be given to 'Anonymous Referee' and the source.
The images or other third party material in this Peer Review File are included in the article's Creative Commons license, unless indicated otherwise in a credit line to the material. If material is not included in the article's Creative Commons license and your intended use is not permitted by statutory regulation or exceeds the permitted use, you will need to obtain permission directly from the copyright holder.
To view a copy of this license, visit https://creativecommons.org/licenses/by/4.0/

The authors would like to thank both the reviewers for the careful reading of the manuscript, for having raised various important issues, and for the valuable and constructive comments. Below, we discuss point-by-point all issues raised by each reviewer (which are reported in blue for convenience). References to modifications introduced in the revised manuscript are displayed in *italics*. All line numbers mentioned refer to the previous version of the manuscript, i.e., the original pdf file of the reviewer, unless specified otherwise. For clarity, some reviewer comments have been subdivided into bullet points to facilitate a more structured response.

We have also revised the manuscript according to the editorial guidelines. This includes:

1. The abstract has been revised.
2. A Plain Summary and an Editorial Summary have been included.
3. The manuscript structure has been changed from Introduction – Results – Discussion – Methods to Introduction – Methods – Discussion – Results.

Reviewer #1

The authors demonstrates the first result of application of PET-guided adaptive radiotherapy in an animal study. This can improve accuracy of dose delivery in particle therapy. The manuscript is well written and illustrated but a few issues should be addressed before publication.

Major comments:

1) The manuscript lacks any discussion of the limitations of the present study, the disadvantages of the proposed strategy, and the potential technical challenges associated with the clinical implementation.

   We thank the referee for pointing this out as also done by the other reviewers. We have therefore substantially revised the *Discussion* section (*pages 17–19 of the new manuscript*) to explicitly address the limitations of the present work and the challenges associated with clinical translation.

   In particular, we now include a dedicated paragraph discussing (i) the restriction of the present proof-of-principle study to a murine model with simplified and relatively homogeneous anatomy compared with patients, (ii) the impact of biological washout and the absence of clinically relevant uncertainties such as respiratory motion, positioning variability, and anatomical heterogeneities, (iii) the reliance of the proposed "aim-and-shoot" workflow on accurate WET estimation and controlled beam delivery conditions, including considerations related to PBS delivery and cumulative PET activity reconstruction, and (iv) the current technological limitations associated with radioactive ion beam production, handling, and clinical implementation, which remain restricted to specialised facilities and therefore may limit near-term clinical translation.

2) Lines 164-165: how many spills and particles in total were delivered to the spot for the confirming the range? The dose of 5 Gy seems significant for probing beams. Can it be reduced and to what value?

   We thank the reviewer for raising such an interesting question. In the present experiment, the range verification beam was delivered through two beam-spots in the y direction (±3 mm), with 3.65×10^5 particles/spot repeated for 500 times for the probing irradiation. However, the effective particle transmission through the beamline is reduced by collimation and shielding elements, and, in the case of probe beam irradiation, by also the collimator with 3 mm diameter. Consequently, not all primary particles delivered contribute to the in-beam PET signal, which was acquired simultaneously with beam delivery during irradiation. Using FLUKA simulations, we quantify a transmission factor of approximately 1% through the full beamline, defined after the final 3 mm collimating aperture and before the compensator.

   The initial choice of a 5 Gy probing dose was based on our previous study, where we demonstrated that such dose does not induce significant tumor damage when delivered in a single fraction to the whole tumor. Therefore, the probing irradiation with a smaller field was considered biologically sub-therapeutic and not expected to bias the subsequent treatment response in the present experiment. The dose rate for the probing irradiation was approximately 1 Gy/min, corresponding to about 5 minutes total beam delivery. We agree that 5 Gy may appear relatively high for a verification procedure but, for this study, we chose to nevertheless collect more statistic to make sure that the activity images had enough statistic to clearly discriminate the ranges while still not affecting the biological response.

   Based on reviewers' suggestion, we now performed an additional analysis and estimated that accurate range detection can be achieved after 25–30 s of beam delivery, corresponding under our experimental conditions to approximately 0.4–0.5 Gy delivered dose, i.e., 3×10^7 delivered particles, with only 3×10^5 particles effectively contributing to the PET signal for range

verification. This indicates that substantially lower probing doses and particles are sufficient for reliable range verification.

We have included this information in the revised manuscript. In particular: (i) the "Irradiation" section (L156 in the previous version), now revised also accordingly to the editorials' requests and renamed "*Treatment Planning and Irradiation*" (*pages 8–9 of the new version of the manuscript*), includes detailed information on the number of particles delivered per spot and the number of rescans required to achieve the 5 Gy probing beam; (ii) the rationale for selecting a 5 Gy probing dose has also been added in the same section; and (iii) the minimum acquisition time and dose/number of particles required for accurate range verification, together with a scatter plot (new *Supplementary Figure 4*) illustrating range detection accuracy during and after irradiation along with the number of counts acquired in the same time with the in-beam PET scanner (new *Supplementary Figure 5*), have been included in the *"PET activity"* section of the Results *(pages 14–16 of the revised manuscript)*.

3) Lines 313-315: how does the signal-to-noise ratio of a PET image relate to particles per spot? Do you have any quantitative data on this? I wonder what minimum beam intensity would allow for range verification. If it is not possible to significantly reduce the intensity of the probing beams, perhaps it can be possible to take them into account when calculating the dose during treatment planning? Please clarify these points in relation to the clinical implementation of your strategy.

The signal-to-noise ratio (SNR) of the reconstructed PET image is directly related to the number of detected annihilation events and therefore to the number of delivered particles per spot. As mentioned in our response to the previous question, in the present work the range verification beam was delivered through two beam-spot in the y direction (±3 mm), with approximately 3.65×10^5 particles/spot repeated for 500 times for the probing irradiation. Nevertheless, our analysis indicates that reliable range verification does not require the full delivered statistics. Approximately 25–30 s of acquisition, corresponding to only about 0.4–0.5 Gy delivered dose under the present experimental conditions. Since PET counting statistics scale approximately with the number of particles reaching the PET scanner and the image SNR follows Poisson behavior, we observed that approximately 3×10^5 particles were already sufficient to identify the distal fall-off with acceptable accuracy for range verification.

Although a systematic determination of the minimum detectable particle intensity was beyond the scope of the present study, the current data indicate that clinically acceptable verification doses well below therapeutic levels are feasible. We have included this information in the "*PET activity*" section of the Results (*pages 14–16 of the revised manuscript*).

Minor comments:

4) Line 143: maybe "0.4 mm" instead of "0.40 mm"

We maintained "0.40 mm" because the zero reflects the measurement precision.

5) Figure 2: arrow for "Compensator" was not provided

Thank you, we now included the correct figure.

Reviewer #2

I would like to commend the authors for this innovative and technically demanding study. The demonstration of real-time in-beam PET for monitoring radioactive ion beam delivery in a pre-clinical mouse model is a valuable contribution to the field, and the methodology and statistical analyses are sound, comprehensive and well-executed.

1) My primary concern relates to the framing of the work. The manuscript implies in several places that this constitutes a demonstration of adaptive radiotherapy, with repeated reference to "real-time" adaptation. However, the study does not appear to include an actual modification of the treatment plan in response to the PET signal. Indeed, the manuscript itself states that the probe beam is used to confirm the range, rather than to determine or adapt the initially planned delivery. What is demonstrated is the feasibility of real-time in-beam PET as a monitoring tool, which is an important and meaningful achievement in its own right, but distinct from real-time adaptive delivery. I would encourage the authors to refocus the manuscript on the added value of PET guidance and its potential role as an enabler of adaptive workflows, rather than presenting the work as a demonstration of a complete adaptive solution. This reframing would, in my view, result in a more accurate and ultimately more compelling narrative.

   We thank the referee for raising this important point. We have fully revised the manuscript to remove references to "real-time treatment adaptation," which could be misinterpreted as clinical replanning within an online-adaptive therapy workflow. Instead, we now consistently use the term "*real-time range-guided*" therapy (see new *References n. 38* and *n. 49* in the new version of the manuscript), as now defined in the *Introduction* ("In this paper, we use the term 'real-time' to describe *dynamical and fast range monitoring and adaptation during irradiation*"). As shown in Supplementary Video 1, new *Supplementary Figure 4* and *Supplementary Figure 5*, the procedure consists of verifying correct dose delivery and iteratively adjusting the beam range until the desired target depth is reached, by an energy change. In our fixed beamline setup, this is achieved by modifying the range shifter (RS) plates to obtain different penetration depths in the mouse model.

   We acknowledge that this represents a really simplified adaptive scenario, involving a small target, a single beam geometry, and a priori knowledge of the water-equivalent thickness (WET). As mentioned before, we therefore refer to our procedure as "*real-time range-guided*" therapy Those limitations have been inserted in the *Discussion* section, *pages 17–19* of the new version of the manuscript. Nevertheless, as also pointed out by the referee, these results demonstrate key enabling capabilities toward future real-time adaptive strategies.

2) Relatedly, the manuscript would benefit from a discussion of what would be required to bridge the gap toward genuine real-time adaptation, including how dose delivered early in a fraction would be accounted for when modifying the remaining plan-to-be-delivered, and what computational and dosimetric infrastructure this would demand. In this context, the probe beam dose of 5 Gy warrants particular attention. This is a substantial dose, and it would be valuable to discuss how this would be managed in a scenario where the PET signal reveals an unexpected discrepancy rather than confirming the expected range. The authors might also consider whether 5 Gy represents the minimum dose required to achieve a usable signal, and whether the approach would remain viable in conventionally fractionated treatment schemes.

   As also in reply to Referee 1, we have substantially revised the *Discussion* section (*pp.17–19*) to explicitly address the limitations and the challenges.

   Regarding the 5 Gy probing dose, this value was based on our previous study, where we demonstrated that such a dose does not induce significant tumor damage when delivered in a single fraction. Moreover, in contrast to the previous study, the volume receiving the 5 Gy probe beam was significantly smaller than the CTV. This information is now included in the revised

“Irradiation” section (L156 in the previous version), now renamed “*Treatment Planning and Irradiation*” (*pages 8–9*) in Methods. While the biological effectiveness of ^{11}C ions is expected to be comparable to stable carbon ions, the comparison between a single 5 Gy exposure and conventionally fractionated treatment schemes (e.g., 16 fx × 3 Gy RBE) is not directly meaningful in this context. Instead, we emphasize that the probe dose is biologically sub-therapeutic and can be further reduced, supporting the feasibility of this approach without compromising normal tissue constraints. Therefore, it does not significantly bias the subsequent therapeutic outcome in this experiment. We acknowledge that 5 Gy may appear relatively high for a verification step; however, it should be noted that this is not intended as a therapeutic dose but as a robust experimental condition in this proof-of-principle study. In future PBS scenarios, the probing beams may be selected from therapeutic spots belonging to the plan.

Accurate range detection, instead, as in reply to Referee 1, can already be achieved using only the first 25–30 s of PET acquisition, corresponding under our experimental conditions to approximately 0.4–0.5 Gy delivered dose. The minimum acquisition time and dose/number of particles required for accurate range verification, together with a scatter plot (new *Supplementary Figure 4*) illustrating range detection accuracy during and after irradiation along with the number of counts acquired in the same time with the in-beam PET scanner (new *Supplementary Figure 5*), have been included in the “*PET activity*” section in Results (*pages 14–16 of the revised manuscript*).

In the case where the PET signal indicates an unexpected range discrepancy rather than confirming the expected range, and within the simplified framework of our experiment, the beam range is first verified within 25–50 s and then adjusted until the target range is reached via an energy change. In our fixed beamline setup, this adjustment is implemented by modifying the range shifter (RS) plates to emulate different penetration depths in the mouse model. With the revised terminology referring to “*real-time range-guided therapy*” we believe this aspect is now more clearly and appropriately framed throughout the manuscript.

3) A further point concerns the delivery system itself. The current implementation is demonstrated on passive beam delivery system, whereas the majority of modern particle therapy facilities use active scanning systems. The authors should discuss the applicability of this approach to scanning-based delivery, and whether any transition back to passive systems would be required to implement such PET guidance based workflows in contemporary clinical settings.

In the case of pencil beam scanning (PBS), the inherently time-structured spot delivery introduces a dynamic irradiation pattern; however, the PET signal integrates the induced activity over the acquisition time window. As a result, the reconstructed PET images represent the cumulative three-dimensional activity distribution rather than the individual spot-by-spot delivery sequence since the transverse spot pattern is spatially averaged over the detector integration time. This has now been explicitly implemented in the *Discussion* section (*pp. 17–19*). Importantly, a recent study has demonstrated the capability of the present PET system to operate under intensity-modulated proton therapy, supporting its applicability to clinically relevant delivery schemes (see Evangelista, F. et al. “First in-vivo IMPT Irradiation of Mice with Quasi-Real-Time Range Verification with the SIRMIO PET Scanner.” 2025 IEEE Nuclear Science Symposium (NSS), Medical Imaging Conference (MIC) and Room Temperature Semiconductor Detector Conference (RTSD) (2025): 1-1.)

4) The manuscript would also benefit from a more candid discussion of limitations, including the scalability of the approach to larger animals and humans, potential confounds on the PET signal such as biological washout, patient motion, and irradiation timing, and the translational relevance of the mouse model more broadly.

As in reply to Referee 1, we have substantially revised the *Discussion* section (*pages 17–19 of the new manuscript*) to explicitly address the limitations of the present work and the challenges associated with clinical translation.

In particular, we now include a dedicated paragraph discussing (i) the restriction of the present proof-of-principle study to a murine model with simplified and relatively homogeneous anatomy compared with patients, (ii) the impact of biological washout and the absence of clinically relevant uncertainties such as respiratory motion, positioning variability, and anatomical heterogeneities, (iii) the reliance of the proposed "aim-and-shoot" workflow on accurate WET estimation and controlled beam delivery conditions, including considerations related to PBS delivery and cumulative PET activity reconstruction, and (iv) the current technological limitations associated with radioactive ion beam production, handling, and clinical implementation, which remain restricted to specialised facilities and therefore may limit near-term clinical translation.

Minor comments:

1) L68–69: The statement that images do not provide information on dose deviations directly is technically correct, but it would be worth acknowledging that dose deviation estimates can nonetheless be derived or inferred from such images. A small rephrasing to reflect this nuance would be helpful.

   We thank the referee to point out this. We have revised the sentence as:
   "However, those imaging techniques do not provide information on potential dose deviations *during irradiation, although such deviations can be indirectly estimated via image-based dose recalculation performed either prior to or following the treatment session*."

2) L140: To improve readability, it would be beneficial to briefly indicate at this point what the two irradiation conditions are, rather than leaving this implicit until later.

   We have revised the text to clarify that the comparison is between pristine and SOBP irradiation (indeed, range modulator not present or present in the beamline) for the same irradiation mode. Below the new version:
   "Owing to the discrete set of absorber plate thicknesses *available and the water equivalent thickness (WET) of the 2DRM*, an exact match in penetration depth *between pristine and SOBP irradiations could not always be achieved for the same irradiation mode*."

3) L165: The manuscript describes the feedback as instantaneous; however, 25 seconds may not meet the threshold typically associated with that term in a clinical or real-time monitoring context.

   We agree that the term "instantaneous" is not appropriate in the context of the current implementation. We clarify this in the text as follows:
   "In-beam PET images (see following Section) were produced in approximately 25 seconds, providing *fast* feedback on the beam range."

   We further clarify that the present system achieves range verification within approximately 25 s, which does not meet the sub-second scale typically associated with prompt feedback in particle therapy [1]. Nevertheless, to the best of our knowledge, this represents a competitive performance among online PET-based monitoring approaches. Indeed, conventional PET-based monitoring relying on long-lived positron emitters intrinsically limits prompt feedback, whereas true real-time (millisecond-scale) verification would require the use of very short-lived isotopes and dedicated fast readout systems [2]. Ongoing developments in detector electronics and data processing, particularly FPGA-based and high-throughput real-time architectures, are expected to further reduce latency and may ultimately enable sub-second or millisecond-scale range verification in future systems (see new *References n. 25* and *n. 44* in the new version of the manuscript and [3-4]).

   For sake of paper length and since it out of the scope of our work we decided to not include this additional information in the manuscript. Below the references cited in this answer:

[1] Arjomandy, B., et al. (2019), AAPM task group 224: Comprehensive proton therapy machine quality assurance. Med. Phys., 46, doi: 10.1002/mp.13622

[2] Ozoemelam I., et al. (2020) Real-Time PET Imaging for Range Verification of Helium Radiotherapy, Front. Phys. 8:565422. doi: 10.3389/fphy.2020.565422

[3] R. J. Aliaga, et al. (2011) PET System Synchronization and Timing Resolution Using High-Speed Data Links, IEEE Transactions on Nuclear Science, vol. 58, no. 4, pp. 1596-1605, doi: 10.1109/TNS.2011.2140130.

[4] Lingyun K., et al. (2021) A real-time sorting algorithm for in-beam PET of heavy-ion cancer therapy device, Nuclear Engineering and Technology, Volume 53, Issue 10, doi: 10.1016/j.net.2021.04.006.

4) L184–189: The suggestion that positional discrepancies necessarily call for online adaptation may be too strong. In conventional clinical practice, such discrepancies would typically first prompt a review of immobilization and positioning protocols, with online adaptation representing one possible but not necessarily the primary response. A more measured phrasing here would better reflect clinical reasoning.

Following our response to Question 1, we agree that the term "online treatment adaptation" was not appropriate in our context. We have therefore revised the manuscript and consistently replaced it with "*real-time range-guided*" therapy.

We also agree with the referee that, in clinical practice, positional discrepancies would typically first prompt verification of immobilization and setup procedures, with online adaptation representing only one of several possible subsequent actions. We have revised the sentence at the end of section "*Treatment planning and Irradiation*", *page 9* of the new version of the manuscript in Methods, accordingly:

"*Our* workflow *follows a range-guided* radiotherapy paradigm, in which imaging acquired immediately prior to irradiation is used for *real-time* in-beam PET *range* verification *and adaptation*. *The importance of pre-treatment anatomical verification* can be seen by comparing Fig. 4C, showing a mouse anatomy on a control µCT in horizontal *position (see section CT imaging – Horizontal imaging)* the day before the irradiation, to Fig. 4D showing the CBCT of the same animal at the time of irradiation. Clearly, the tumor is shifted away from the irradiation field. *This would require not range adjustment (at least not in the first place) but instead a vertical adjustment of the mouse holder to bring the tumor in the irradiation field, which was not technically possible since the setup was fixed*."

Reviewer #3

The manuscript presents a novel in vivo study investigating whether radioactive ion beams can support in-beam PET-guided adaptive particle therapy. Using an osteosarcoma C3H mouse model, the authors acquired CT images in the same vertical position used for irradiation and visualized a therapeutic β+-emitting ^11C beam in real time with the SIRMIO in-beam PET scanner. Three predefined beam depth settings were tested, corresponding to short, right, and long ranges, with the goal of identifying the "Goldilocks" beam position that adequately covers the tumor while minimizing dose to surrounding normal tissues. In addition to the imaging component, the study also evaluates tumor response and normal tissue toxicity across the different beam range conditions. The authors show that different beam positions generate spatially resolved PET signals associated with distinct biological outcomes, and they propose that radioactive ion beams may enable real-time adaptive treatment. Overall, the work is creative, technically interesting, and potentially important because it moves this concept into an in vivo setting and suggests a path toward PET-guided range assessment in particle therapy.

A. My main concern is that the manuscript may currently overstate the degree of "real-time adaptive therapy" demonstrated. Based on the described short/right/long beam design, the work appears to provide a strong in vivo proof-of-concept for PET-guided beam range assessment or range selection, but not yet a fully realized closed-loop adaptive radiotherapy workflow as many readers may interpret that term. The authors should therefore define much more clearly what was adapted, when the adaptation occurred, and whether the in-beam PET signal was used prospectively during the same experiment to alter treatment delivery.

   We thank the referee for this important clarification, which is also consistent with the comments from the other reviewers. We agree that the term "real-time adaptive therapy" is not appropriate. We have therefore revised the manuscript accordingly and consistently replaced it with "*real-time range-guided*" therapy (see new *References n. 38 and 49* in the revised manuscript) as defined in the *Introduction*.

   Importantly, we now explicitly clarify that the in-beam PET information was used prospectively within the same experimental irradiation to guide beam range selection, but in a simplified and controlled pre-clinical setting rather than a full clinical adaptive replanning framework. As shown in Supplementary Video 1, new *Supplementary Figures 4 and 5*, the procedure consists of verifying correct dose delivery and iteratively adjusting the beam range via energy changes until the desired target is reached. In our fixed beamline configuration, this is implemented by modifying the range shifter (RS) plates to achieve different penetration depths in the ex vivo mouse model.

   We further emphasize that this represents a proof-of-concept scenario with significant simplifications, including a small target volume, lack of severe heterogeneities, a single beam geometry, and prior knowledge of the WET, which limits direct clinical interpretation as a full adaptive workflow. These limitations are now explicitly discussed in the revised manuscript (*Discussion, pp. 17–19*).

B. In addition, the "Goldilocks range" concept is appealing but needs a clearer quantitative decision criterion, including how the optimal range was determined from the PET signal and how reproducible that determination was across animals.

   We have now revised the "Irradiation" section, now "*Treatment planning and Irradiation*" in Methods, including further details regarding our range verification approach:
   "*From our previous experiments, we established that the position of the 80% distal dose fall-off (R80) can be derived from the PET activity peak, enabling us to perform accurate beam range determination in each irradiation mode. Further estimation of R60 and R95 reference depths were derived from monoenergetic beam dosimetry in water (see Methods), where they were found to lie*

approximately +1 mm and −1.5 mm relative to R80, respectively. We further verified through simulations that these millimetric offsets are also reproduced in the activity distributions, which consistently exhibit the corresponding spatial shifts along the beam axis in the PET images. This spatial relationship was used for initial verification of each beam range (S, R or L) by observing the in-beam PET activity distributions obtained during the irradiation with the collimated monoenergetic ^{11}C beam, overlapping them with the CBCT scans of the respective animals."

C. The manuscript would also benefit from a more careful discussion of the known mismatch between PET activity and actual dose distribution, including whether and how the radioactive ion beam approach mitigates this limitation.

We acknowledge the referee and agree that a clearer discussion of the mismatch between PET activity and the actual dose distribution is important. While this aspect was briefly mentioned in the first part of the Introduction, we have now revised the section to include a more explicit and structured discussion to better contextualize our approach and highlight its advantages for accurate, real-time range verification and adaptation (see *page 5* of the new manuscript version).

D. Finally, some of the language, particularly the suggestion that radioactive ion beams may be the "ideal tool," feels stronger than the current data can fully support and should be toned down unless justified with broader comparison and validation.

We have revised the text as:

"Here, we *investigate* the hypothesis that radioactive ion beams *may provide an effective platform for real-time range-guided particle therapy through in-beam PET-based range verification with sub-millimetric precision (<0.5 mm)*"

Specific comments,

1) Line 157, it was not clear how the three plans (S, R and L) were created. Is it based on a previous CT scan for the animal? And the compensator was generated through some planning process to match with the particular anatomy site you plan to treat?

Thank you for highlighting this. The three treatment plans (S, R, and L) were generated based on previously acquired SARRP CT scans of the animals, obtained in the context of our earlier study. From those data, we verified that anatomical variations between different mice along the beam direction were within the spatial resolution of the SARRP system (i.e., below 0.3 mm). Therefore, no significant anatomical discrepancies were expected to affect the treatment plans and the irradiation of the individual animals during the experiment. The treatment plans were then generated using the FLUKA Monte Carlo code, by using the previously validated beam model, the SARRP CT datasets of the animals, and the beamline components.

We have revised the corresponding sections of the manuscript (*Methods, Treatment planning and Irradiation, pages 8−9*) to better clarify this workflow and improve the description of plan generation.

With regard to the compensator, we included more details about it in the "*Radioactive ion beam production and delivery*" section, *pages 6−7*:

"*The mice were treated in a vertical position inside the SIRMIO PET scanner (see section PET imaging for details), fixed onto a custom-made holder (compensator) in a way that the center of the generalized clinical target volume was located at the isocenter position of the PET scanner. In particular, the inner structure of the compensator was anatomically shaped to ensure reproducible mouse positioning, while the outer surface included a 5 mm-deep dimple corresponding to the WET up to the distal edge of a universal target region (named generalized CTV) that accounts for small variations in tumor size and location between individual animals*."

2) Line 164, how long is the delivery for the first 5Gy spot? Is the PET concurrent with the delivery or after the delivery? How did you decide to choose 5Gy? It seems to be quite high dose that choose for the range calibration purpose.

The delivery time for the initial 5 Gy spot is approximately 5 minutes. PET acquisition is performed concurrently with beam delivery in an in-beam PET configuration, enabling continuous monitoring during irradiation. The 5 Gy value was initially selected based on our previous study, where we demonstrated that such dose delivered to the full CTV volume in a single fraction did not induce significant tumor damage and were sufficient to collect good quality PET images.

Importantly, we now clarify in the manuscript that significantly lower doses are in fact sufficient for range determination. Based on the present data, approximately 25s of acquisition already provides adequate statistics to identify the beam range, corresponding to a delivered dose of about 0.4 Gy under our experimental conditions. This indicates that, in a clinical implementation, the dose required for range verification could be kept well below therapeutic levels.

3) Line 166, how did you confirm the range from the probe irradiation? Is there a particular location you're comparing with?

As in response to Question 1, we revised the "*Treatment Planning and Irradiation*" section in Methods, *pages 8−9*, by including: (i) range (S, R, L definition), based on Monte Carlo–derived depth–dose calculations. (ii) the previously established correspondence between the PET activity peak and the distal 80% dose fall-off (R80) was used to infer beam range during irradiation, with R60 and R95 derived from calibrated monoenergetic dosimetry and confirmed by simulations of the activity distributions. This revised structure clarifies the planning basis, the simulated dose–range correspondence, and how PET-based range verification was used during the irradiation workflow.

4) Line 176, does the subject stay at the same location between the CBCT image and in-beam PET?

The SARRP device was located in the same room as the BARB beamline, only 1-2 meters away from it. Before the CBCT, the mouse was positioned on the gas-tight irradiation bed, which, after the scan acquisition, was replugged from the SARRP to the SIRMIO platform, without awaking and repositioning the animal on the holder, therefore preserving its anatomy between the CBCT scan and the irradiation. We included this information in the "*Treatment Planning and Irradiation*" section, *page 9*, as follows:

"Immediately before the start of the treatment, we acquired a CBCT of each mouse using the Small Animal Radiation Research Platform (SARRP)*, see Fig. 4A and section CT imaging – Vertical imaging,* to get the anatomical image of the animal in exactly the same vertical position used for irradiation (Fig. 4B). *This was possible due to using the custom-made holder compatible with both setups, avoiding awaking and repositioning the animal between the CBCT scan and irradiation.*"

5) Line 187, it's quite confusing that you claim adaptive workflow but the workflow cannot handle anatomy change between the μCT and delivery day CBCT.

There might be a confusion coming from using the term "adaptive", that now have been replaced with *real-time range-guidance*. In our setup and throughout the manuscript, this means the fast verification and adjustment of the beam range in case of observed discrepancies between the observed and predicted activity peak positions. This would be the case if, for example, the tumor demonstrates the significant growth between the uCT and delivery day CBCT, or the curvature of the spine changes, affecting the overall mouse anatomy (as noticed in our previous study). The case presented in Figure 4 instead illustrates the other anatomical issue where the tumor has moved in the superior-inferior direction and went out of the planned irradiation field. This would have required not the range adjustment (at least not in the first place) but instead the vertical adjustment of the mouse holder to bring the tumor in the irradiation field, which was not technically possible since the setup was fixed horizontally.

As in reply to Referee 2, we know that in clinical practice, positional discrepancies would typically first prompt verification of immobilization and setup procedures, with online adaptation representing only one of several possible subsequent actions. We have revised the sentence at the end of section "*Treatment planning and Irradiation*", in *page 9* of the new version of the manuscript, accordingly.

6) Line 196, it would be better to have a quantitative comparison between the position of activity peak and the 80% distal fall off position. Also it is not clear how many animals were used in the experiment for a statistical analysis.

Thank you for pointing out this. The 80% fall-off, as mentioned above, is based on our previous experiment. We have revised the "*Introduction*" to better contextualize our approach and highlight its advantages for accurate, real-time range verification, as well as "*Treatment planning and Irradiation*", *pages 8−9* (see response 3 above).

With regards to the animal numbers, the initial group sizes for S, L and R groups were identified in the end of the "*Animal model*" section of the "*Methods*" part. For the tumor growth analysis and the normal tissue toxicities, the group sizes and the reasons for them to differ from the initial group sizes are indicated in the respective figure captions (Figures 8 and 9, respectively).

7) Line 300, for general adaptive purposes, beside the distal range information, one still need lateral profile info and spot intensity info. It's not clear how that information can be obtained with in-beam PET images.

We thank the referee for this insightful comment. We agree that, in a full adaptive framework, lateral dose profile and spot intensity information are important for adaptive therapy. We have revised the manuscript to remove references to "real-time adaptation" and consistently use the more accurate term "*range-guided therapy*" where the current work focuses primarily on longitudinal range assessment and adaptation.
